# Antimony-doped graphene nanoplatelets

**DOI:** 10.1038/ncomms8123

**Published:** 2015-05-22

**Authors:** In-Yup Jeon, Min Choi, Hyun-Jung Choi, Sun-Min Jung, Min-Jung Kim, Jeong-Min Seo, Seo-Yoon Bae, Seonyoung Yoo, Guntae Kim, Hu Young Jeong, Noejung Park, Jong-Beom Baek

**Affiliations:** 1 School of Energy and Chemical Engineering/Low-Dimensional Carbon Materials Center, Ulsan National Institute of Science and Technology (UNIST), Banyeon 100, Ulsan 689-798, South Korea; 2 School of Natural Science/Low-Dimensional Carbon Materials Center, Ulsan National Institute of Science and Technology (UNIST), Banyeon 100, Ulsan 689-798, South Korea; 3 UNIST Central Research Facilities (UCRF), Ulsan National Institute of Science and Technology (UNIST), Banyeon 100, Ulsan 689-798, South Korea

## Abstract

Heteroatom doping into the graphitic frameworks have been intensively studied for the development of metal-free electrocatalysts. However, the choice of heteroatoms is limited to non-metallic elements and heteroatom-doped graphitic materials do not satisfy commercial demands in terms of cost and stability. Here we realize doping semimetal antimony (Sb) at the edges of graphene nanoplatelets (GnPs) via a simple mechanochemical reaction between pristine graphite and solid Sb. The covalent bonding of the metalloid Sb with the graphitic carbon is visualized using atomic-resolution transmission electron microscopy. The Sb-doped GnPs display zero loss of electrocatalytic activity for oxygen reduction reaction even after 100,000 cycles. Density functional theory calculations indicate that the multiple oxidation states (Sb^3+^ and Sb^5+^) of Sb are responsible for the unusual electrochemical stability. Sb-doped GnPs may provide new insights and practical methods for designing stable carbon-based electrocatalysts.

## Abstract (web summary)

Heteroatom doping of graphitic networks has been shown to modify electrocatalytic performance. Here, the authors fabricate semimetal antimony doped graphene nanoplatelet based catalysts and demonstrate their enhanced stability for oxygen reduction, due to the multiple accessible antimony oxidation states.

## Graphical abstract

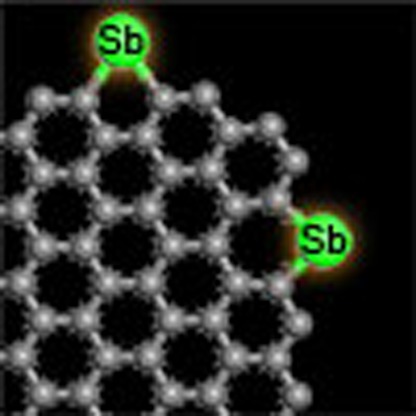

Energy demand is continually increasing. However, fossil fuel supplies are declining, and levels of greenhouse gases are rising due to rapid industrialization and population growth. The majority of energy demand is still met by conventional fossil fuels, such as coal, oil and natural gas. Now, human beings are facing a shortfall in natural energy resources and environmental pollution. Therefore, the search for alternative renewable energy resources, such as solar, wind, thermal, hydroelectric, biomass and fuel cells^1^,^2^,^3^, has received tremendous research interest. Fuel cells are considered one of the most promising renewable energy candidates after NASA (National Aeronautics and Space Administration) chose them as the primary power supply for the Apollo spacecraft in the early 1960s (ref. 4). Fuel cells possess many advantages over batteries, such as energy security, low operating cost, stable power generation, fuel choice, clean emissions and high power efficiency^5^. However, two major technical pitfalls: manufacturing cost and reliability; have hampered their commercialization to date. Specifically, the major drawback is the kinetically slow cathodic oxygen reduction reaction (ORR) compared with the fast anodic hydrogen oxidation reactions^6^,^7^,^8^. Platinum (Pt)-based materials are the most efficient catalysts discovered so far. However, in addition to their high manufacturing costs due to the scarcity of Pt, these fuel cells suffer from a variety of problems associated with carbon monoxide poisoning, fuel selectivity and long-term stability. Therefore, developing low-cost, fuel-selective, durable and highly active electrocatalysts for large-scale commercial applications is a considerable challenge. As a result, significant efforts have been devoted to finding possible alternatives to Pt, including Pt-based alloys^9^,^10^, nonprecious metal-based catalysts^11^,^12^, enzymatic electrocatalysts^13^ and heteroatom-doped carbon-based materials^14^,^15^,^16^,^17^,^18^,^19^. Among these candidates, heteroatom-doped carbon-based materials, such as carbon black^20^, carbon nanoparticles^21^, carbon nanotubes^14^ and graphene^16^, have been intensively studied as efficient metal-free electrocatalysts for the ORR.

Graphene, which is a carbon-based material consisting of a single layer of *sp*^*2*^ carbon with a two-dimensional honeycomb lattice, has demonstrated many peculiar properties, including superior electrical conductivity^22^, large surface area^23^, excellent mechanical flexibility^24^ and high thermal/chemical stability^25^. Therefore, there have been numerous reports on graphene and graphene-related materials doped with various heteroatoms such as boron (B)^18^,^26^, nitrogen (N)^16^,^27^, sulphur (S)^28^,^29^, phosphorus (P)^17^, iodine (I)^19^, selenium (Se)^30^ and their mixtures^31^,^32^. In general, chemical vapour deposition^33^,^34^ and graphite oxide^35^ approaches have been utilized for the synthesis of heteroatom-doped graphene and graphene nanoplatelets (GnPs). However, the approaches are neither cost-effective nor industrially viable due to complex manufacturing procedures and use of hazardous reagents (for example, strong acids and carcinogenic-reducing agents), limiting their potential for commercialization. A long lifetime is the most critical factor for commercialization because almost all electrocatalysts, including Pt/C and heteroatom-doped carbon-based materials, are associated with electrochemical corrosion (etching) of carbon by oxygen into carbon dioxide^36^. Electrochemical etching of carbon (C+O_2_→CO_2_) further impedes the realization of energy conversion and storage devices with long lifetimes and commercial viability. We were able to devise a simple yet eco-friendly mechanochemical reaction for an efficient edge-selective functionalization of GnPs by ball-milling. For example, various functional groups and/or non-metallic heteroatoms were introduced at the edges of GnPs^29^,^37^,^38^.

Here, on the basis of well-optimized ball-milling conditions we demonstrate the doping of metalloid antimony (Sb) at the edges of non-metallic GnPs, that is, the formation of C–Sb bonds (Supplementary Methods). The edge-selectivity of SbGnPs and their stability were visually confirmed using atomic-resolution transmission electron microscopy (AR-TEM). The resultant SbGnPs display enhanced ORR performance, suggesting potential new insights for the design and synthesis of low-cost and highly durable electrocatalysts in fuel cells.

## Results

### Synthesis and characterization of SbGnPs

**Figure 1:**
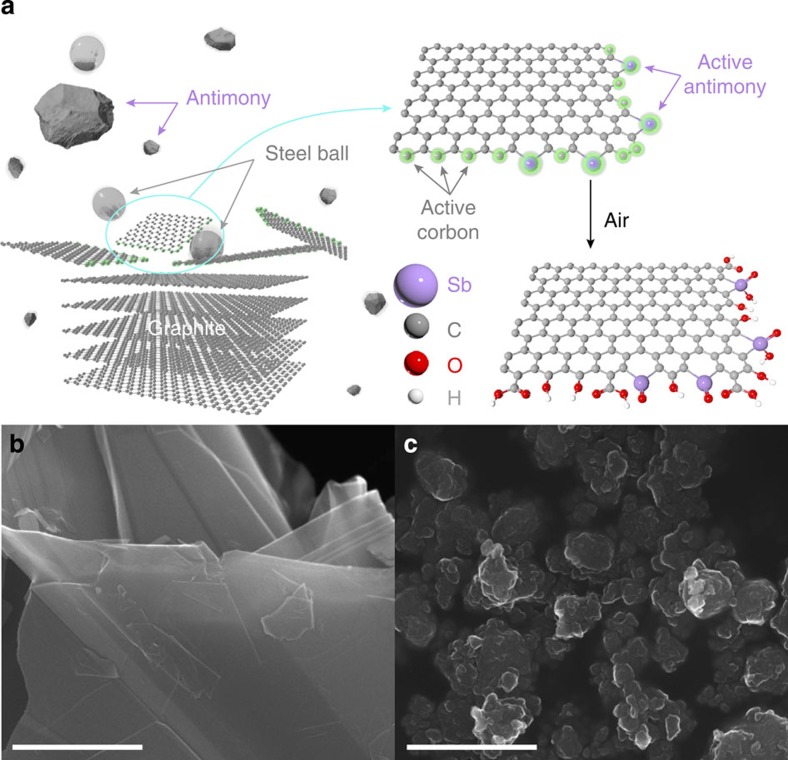
Preparation and morphology of SbGnPs. (**a**) Schematic of mechanochemical ball-milling-driven doping antimony (Sb) to the edges of graphene nanoplatelets (GnPs). SEM images of (**b**) the pristine graphite before ball-milling, (**c**) SbGnPs after ball-milling graphite in the presence of solid Sb and complete work-up procedure, showing the dramatic grain size reduction of SbGnPs (<1 μm) compared with the pristine graphite (<150 μm). Scale bars, 1 μm.

As schematically represented in Fig. 1a, the SbGnPs were prepared by ball-milling graphite in the presence of solid Sb in a ball-mill crusher. Scanning electron microscopy (SEM) images show that the grain size of the pristine graphite (<150 μm) (Fig. 1b) was significantly reduced to <1 μm for SbGnPs (Fig. 1c). This result implies the mechanochemical unzipping of C–C bonds in the graphitic structure and cracking of the Sb crystal generates active carbon and Sb species, respectively, to form C–Sb bonds at the broken edges of the GnPs. Prior to sample characterization, a careful work-up procedure was developed to completely remove the free-standing Sb. The detailed method is amply described in the experimental section in Supplementary Note 1. The TEM analyses with element mappings and the corresponding energy-dispersive X-ray spectrometry (EDS) spectra (Supplementary Figs 1–7) suggested that there were no traces of Cr and Fe residues, whereas Sb was still observed, indicating the formation of C–Sb bonds^39^. The content of Sb in the SbGnPs from the SEM EDS is 1.17 at.% (10.28 wt.%, Supplementary Fig. 8 and Supplementary Table 1).

**Figure 2:**
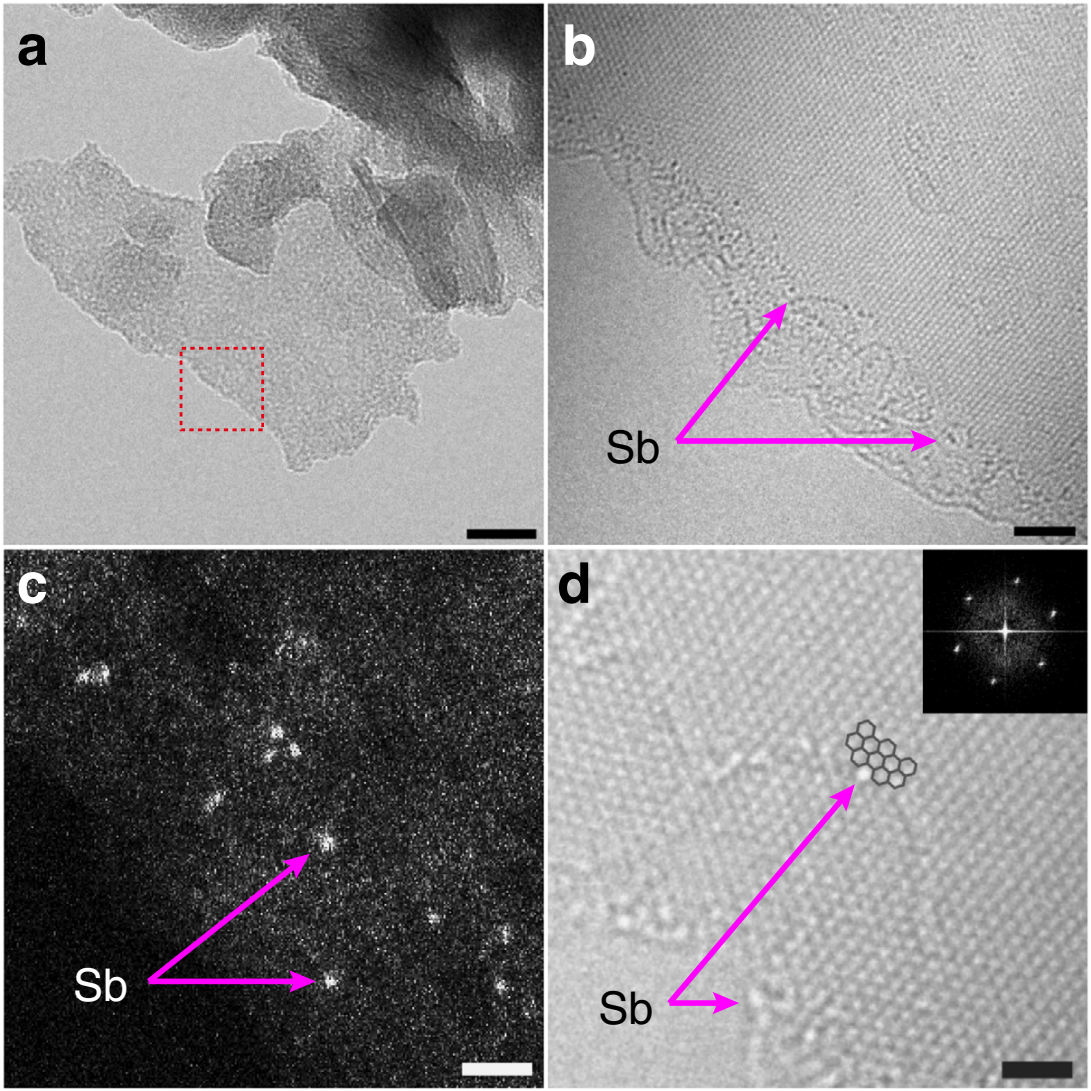
Structural identification of SbGnPs at the atomic level. (**a**) Bright-field (BF) TEM image of SbGnPs after ball-milling. Scale bar, 20 nm; (**b**) Atomic-resolution (AR) TEM image obtained from the region marked with a red square. Scale bar, 2 nm; (**c**) HAADF STEM image showing the contrast of single Sb atoms at the edges of SbGnPs. Scale bar, 1 nm; (**d**) AR-TEM image of a single Sb atom attached at the graphene edge with an armchair configuration. The inset is the fast Fourier transform (FFT) of the image. Scale bar, 1 nm.

Spherical aberration (Cs)-corrected TEM and scanning transmission electron microscopy were employed to prove the edge-selective formation of C–Sb bonds^39^. The AR-TEM images (Fig. 2b and Supplementary Fig. 9b) obtained at the edges of a thin platelet shown in the square of the bright-field (BF) TEM image (Fig. 2a and Supplementary Fig. 9a) clearly show that the Sb atoms with the dark atomic contrast are only observed along the edge lines of SbGnPs. In contrast, the inner part of the sheets shows a perfect hexagonal-lattice image of graphene (Supplementary Fig. 9c,d), indicating that the mechanochemical unzipping of C–C bonds in graphitic structure preserves crystallinity at the basal plane. High angle annular dark field scanning transmission electron microscopy image (Fig. 2c) with atomic Z-contrast also confirms the existence of Sb single atoms (arrows) and not the formation of Sb nanoparticles. It is remarkable that the single Sb atom (bright atomic contrast of Fig. 2d) is attached at the edge of the armchair configuration of graphene, which is in agreement with our selective edge-formation model of C–Sb bonds proposed in Fig. 1a. The TEM results support the notion that ball-milled graphite and solid Sb generate active C and Sb atoms for the formation of C–Sb bonds along the broken edges of GnPs. It is also interesting that the Sb atoms attached to graphene edges are relatively stable and do not catalyse to dissociate the graphitic C–C bonds during the e-beam irradiation (Supplementary Movie 1), compared with general Cu-mediated etching of chemical vapour deposition graphene (Supplementary Movie 2). The stability of C–Sb bonds under e-beam irradiation could also be attributed to the long-term stability of electrochemical performance (*vide infra*).

**Figure 3:**
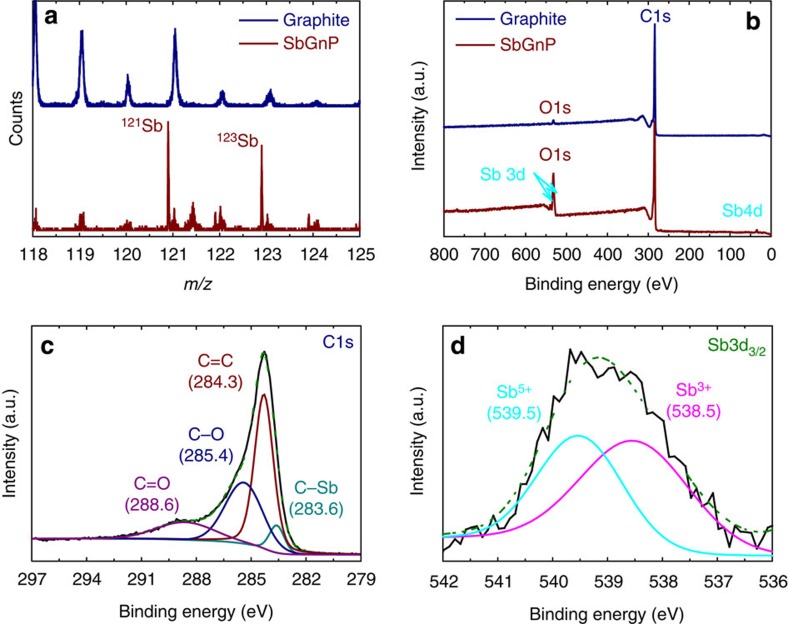
Spectroscopic analysis of the pristine graphite and SbGnPs. (**a**) Time-of-flight secondary ion mass (TOF-SIM) spectra, showing the presence of Sb isotopes (*m/z*=121 and 123) in SbGnPs. XPS survey spectra: (**b**) full spectra, (**c**) C1s and (**d**) Sb3d_3/2_, showing the ratio between Sb^3+^ and Sb^5+^ to be ∼1 and suggesting that the population of proposed structures Sb^3+^ and Sb^5+^ in Supplementary Fig. 12 is similar.

Time-of-flight secondary ion mass spectrometry (TOF-SIMS) was performed on the pristine graphite and SbGnPs to provide further evidence for the presence of Sb in SbGnPs. As shown in Fig. 3a, the positive ion spectrum of SbGnPs indicated the presence of ions ^121^Sb^+^ (*m/z*=121) and ^123^Sb^+^ (*m/z*=123) as isotopes of Sb, whereas the pristine graphite did not exhibit any Sb-related peaks.

Elemental analyses indicated that the pristine graphite had carbon and oxygen contents of 99.64 wt.% and 0.13 wt.% (Supplementary Table 1), respectively. The SbGnPs contained C, H and O of 73.44 wt.%, 0.93 wt.% and 11.95 wt.%, respectively, suggesting that the remainder would be 13.68 wt.% of Sb (Supplementary Table 1). The significantly increased oxygen content in SbGnPs compared with the pristine graphite could be due to the oxidation of remnant active carbon and Sb species with oxygen/moisture in the air on opening the lid of the ball-mill container (see Fig. 1a)^37^,^38^.

Because the boiling point of Sb is as high as 1,587 °C (ref. 40), the Sb content in SbGnPs could be further quantitatively estimated using thermogravimetric analyses (TGAs) in air. The char yield of the pristine graphite at 1,000 °C was 23.7 wt.% (Supplementary Fig. 10a). The high thermal stability of the pristine graphite is due to the large grain size (<150 μm) and therefore reduced number of edges. However, SbGnPs had a maximum weight loss at ∼400 °C and reached the steady state >500 °C. The char yield at 1,000 °C was 10.7 wt.% (Supplementary Fig. 10a), which should originate from the Sb residue and corresponds well with the EDS result (10.28 wt.%, Supplementary Table 1). The char yields of the pristine graphite and SbGnPs at 1,000 °C under a nitrogen atmosphere were 99.1 wt.% and 77.2 wt.%, respectively (Supplementary Fig. 10b). To prove that the residue at 1,000 °C is due to Sb and the relatively lower thermal stability of GnPs is attributed to smaller grain size, other GnPs functionalized with non-metal elements, such as edge-carboxylated^37^, edge-sulfonated^38^ and edge-aminated GnPs^37^, were prepared by ball-milling at similar conditions using the same work-up procedures. They all indicated that the char yields at 1,000 °C approached 0.0 wt.% (Supplementary Fig. 10c,d) and have relatively low thermal stabilities, which resulted from the smaller grain size (the additional edges) by mechanochemical cracking and decomposition of the functional groups at the edges. Therefore, the char yield (10.7 wt.%) of SbGnPs at 1,000 °C is due to the formation C–Sb bonds. In addition, the Sb content quantified using TGA corresponded well with those from SEM EDS (1.17 at.% and 10.28 wt.%; Supplementary Table 1).

X-ray photoelectron spectroscopy (XPS) has been used to further elucidate the formation of C–Sb bonds in SbGnPs (Fig. 3b). The survey spectrum of the pristine graphite exhibits strong C1s and weak O1s peaks. Because XPS is more sensitive to the surface chemical composition^41^,^42^, the O1s peak should be contributed by physically adsorbed oxygen on the surface of graphite. In addition to the C1s and O1s main peaks, the survey spectrum of SbGnPs clearly shows Sb4d and Sb3d peaks^43^,^44^. The Sb content is 1.18 at.% (10.53 wt.%, Supplementary Table 1), which corresponds well with SEM EDS results. The Sb3d peak overlaps with the O1s peak. High-resolution XPS survey spectra, together with the curve fittings and deconvolutions, indicate that the C1s peak consisted of C=O (288.4 eV), C–O (285.2 eV), *sp*^2^ C–C (284.2 eV) and C–Sb (283.6 eV) (Fig. 3c). The Sb3d and O1s peaks divided into Sb3d_5/2_ (530.4 eV) and Sb3d_3/2_ (539.2 eV) peaks as well as C=O (532.4 eV) and C–O (533.7 eV) (Supplementary Fig. 11). In addition, the Sb3d_3/2_ peak (centred at 539.2 eV) can be separated into two contributions: Gaussian lines centred at 538.5 and 539.5 eV correspond to Sb with an oxidation state of Sb^3+^ and Sb^5+^, respectively (Fig. 3d), supporting the structure presented in Fig. 1a (refs 45, 46). The ratio of Sb^3+^ to Sb^5+^ is ∼1, indicating that the presence of Sb in SbGnPs is in the form of the structure proposed in Supplementary Fig. 12 (ref. 47). Because the dangling C–Sb bonds may not be sufficiently stable to withstand a harsh work-up procedure (boiling concentrated hydrochloric acid treatment), the remaining edge structures could be confined to four possible structures (Supplementary Fig. 13). Based on the XPS analysis (Fig. 3d), the populations of Sb^3+^ (Supplementary Fig. 13a.b) and Sb^5+^ (Supplementary Fig. 13c,d) are similar. The structures are the results of zigzag and armchair cuttings, each type of edge generates Sb^3+^ and Sb^5+^. The formation of the structure could be rationalized by understanding Group VA in the periodic table. This group includes N, P, arsenic (As) and Sb, whose relative covalent atomic radii are 70 pm, 110 pm, 120 pm and 141 pm, respectively (Supplementary Fig. 14). The elements P, As and Sb have relatively large atomic sizes, and thus they can accommodate more chemical bonds to form stable structures (pink circles, Supplementary Fig. 14), whereas smaller N cannot. Therefore, like P and As^46^, Sb must be highly susceptible to the highest oxidation state due to oxygen, and Sb^5+^ should be the most preferred form. As a result, the capability of Sb^5+^ formation in SbGnPs are expected to attribute to electrochemical stability, specifically in the presence of oxygen involved reactions, such as the ORR.

The structures of the pristine graphite and SbGnPs were further studied using Raman spectroscopy. The pristine graphite exhibits a sharp G peak due to the well-ordered graphitic structure, and the ratio of the D-band to the G-band intensities (*I*_D_/*I*_G_) was nearly zero (Supplementary Fig. 15a). However, the G-band of SbGnPs at 1,588 cm^−1^ was located at a higher frequency than that of the pristine graphite at 1,585 cm^−1^ due to the concentration of defects, which change from the graphite crystal to a few graphitic layers and the resonation of isolated double bonds^48^. Furthermore, SbGnPs exhibit a stronger D-band intensity at ∼1,350 cm^−1^ and a higher *I*_D_/*I*_G_ ratio of 0.80 due to the edge contribution from grain size reduction (<1 μm, see Fig. 1c) and edge distortion by Sb functionalization (see Fig. 1a).

The degree of exfoliation in the solid state could be estimated by comparing X-ray diffraction patterns (Supplementary Fig. 15b). The pristine graphite exhibits a strong sharp [002] peak at 26.5°, which corresponds to a large number of graphitic layers with an interlayer *d*-spacing of 0.34 nm^49^. The [002] peak for SbGnPs shifted slightly to 24.1° with a negligibly low intensity (0.08% compared with that of the pristine graphite), indicating that the solid-state delamination of graphitic layers into a few layers of SbGnPs that occurred during ball-milling induced functionalization of Sb at the broken edges of the graphitic layers and grain size reduction by cracking. Furthermore, by assuming that SbGnPs have minimal basal plane distortion as the proposed structure (see Fig. 1a) and confirmed by AR-TEM (see Fig. 2b), the Brunauer–Emmett–Teller (BET) surface area can also be conveniently correlated to the average number of graphitic layers in SbGnPs. For example, the maximum BET surface area of a single layer of graphene is 2,630 m^2 ^g^−1^, and the edge contribution to the BET surface area is negligible. Then, the average number of SbGnPs could be estimated in a straightforward manner. First, the BET surface area (2.78 m^2 ^g^−1^, Supplementary Fig. 15c and Supplementary Table 2) of the pristine graphite was significantly increased after ball-milling to produce SbGnPs (376.71 m^2 ^g^−1^, Supplementary Fig. 15d and Supplementary Table 2). The surface area and pore volume of SbGnPs were increased by 136-fold and 241-fold, respectively (Supplementary Table 2), indicating an effective edge delamination due to cracking graphitic layers and doping bulky Sb at the edges of SbGnPs (Supplementary Fig. 16). The average expected number of graphitic layers in SbGnPs was 2,630/376.71, which is equal to ∼7.1 in the solid state and will be further delaminated on dispersion in proper solvents. Together with the high oxidation stability of Sb at the edges of SbGnPs, their large surface area and pore volume are expected to significantly contribute to the electrocatalytic performance.

### Electrocatalytic activity of SbGnPs

**Figure 4:**
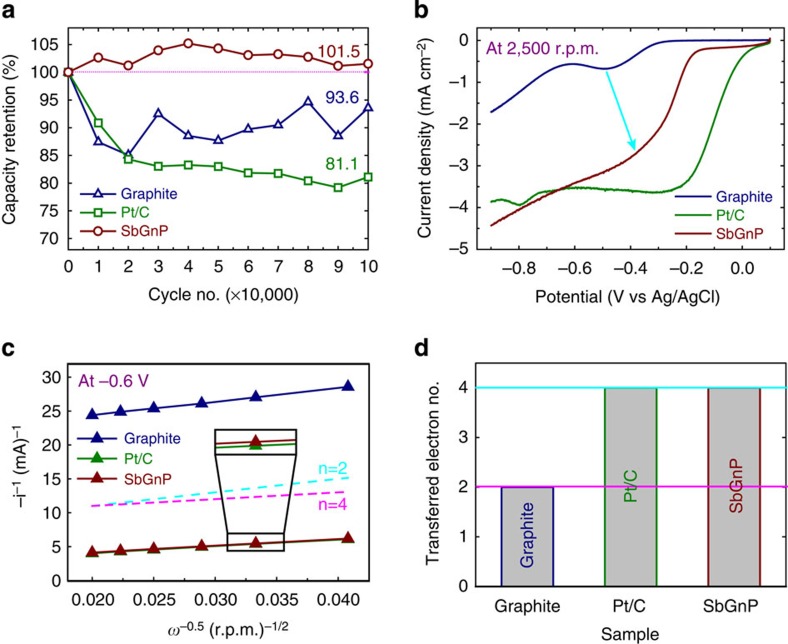
Electrochemical analysis. Electrochemical analysis of the pristine graphite, Pt/C and SbGnPs. (**a**) Capacity retention in an O_2_-saturated 0.1 M aq. KOH solution with a scan rate of 100 mV s^−1^, illustrating that SbGnPs displayed no capacity loss after 100,000 cycles; (**b**) Linear sweep voltammograms (LSVs) at a rotation rate of 2,500 r.p.m. and a scan rate of 10 mV s^−1^; (**c**) Koutecky–Levich plots derived from the rotating disk electrode measurements at −0.6 V; (**d**) The electron transfer number at −0.6 V (versus Ag/AgCl).

After structural identification, the electrocatalytic activity of SbGnPs was first examined using cyclic voltammetry in a nitrogen- and oxygen-saturated 0.1 M aq. KOH solution (Supplementary Fig. 17 and Supplementary Table 3). The pristine graphite and commercial Pt/C were also measured for comparison. Despite the featureless voltammetric currents in the N_2_-saturated medium, well-defined cathodic peaks emerged in the O_2_-saturated medium (Supplementary Fig. 17a–c). The peaks for the pristine graphite, Pt/C and SbGnPs are centred at −0.40 V, −0.23 V and −0.27 V, respectively, with corresponding current densities of −0.23 mA cm^−2^, −0.69 mA cm^−2^ and −0.68 mA cm^−2^, respectively. The results indicated that doping Sb onto graphitic structures not only shifts the cathodic reduction peak more positively (∼0.13 V compared with that of the pristine graphite) but manifests a high electrocatalytic activity. The current density of SbGnPs increased by 296% compared with that of the pristine graphite and equivalent to commercial Pt/C. More importantly, the prolonged cycle stability of SbGnPs was superior to those of the pristine graphite and commercial Pt/C (Fig. 4a and Supplementary Fig. 17d–f). The SbGnPs exhibited no capacitance loss, even after 100,000 cycles in an O_2_-saturated 0.1 M aq. KOH solution with a scan rate of 100 mV s^−1^, whereas the pristine graphite and Pt/C exhibited substantial capacitance losses (6.4% and 18.9%, respectively). Therefore, Sb doping at the edges of graphitic framework plays a pivotal role in the improvement of the overall ORR performance.

To gain further insight into the electrochemical kinetics of the SbGnPs during the ORR process, rotating disk electrode measurements were also performed in an O_2_-saturated 0.1 M aq. KOH solution at various rotating speeds and at a constant scan rate of 10 mV s^−1^. As shown in Supplementary Fig. 18a–c, the diffusion current densities increased with increasing rotation rates, whereas the onset potentials remained nearly constant. Once again, the limiting current density for SbGnPs is considerably higher than those for the pristine graphite and Pt/C at any constant rotation rate at −0.9 V (Fig. 4b). It is well-known that the number of electron transfer (*n*) per O_2_ molecule involved in the ORR process can be calculated from the slope of the Koutecky–Levich plots (Supplementary Fig. 18d–f). As shown in Fig. 4c,d, the experimentally determined *n* value at the potential of −0.6 V for Pt/C exhibited an ideal one-step, four-electron pathway (*n*=4.0) for the ORR. The corresponding numbers of electrons transferred per O_2_ molecule for the pristine graphite and SbGnPs were calculated to be 2.0 and 4.0, respectively (Supplementary Table 4). The pristine graphite exhibits a nearly two-step, classical two-electron process (*n*=2.0) for ORR. In contrast, the ORR process in the SbGnPs is considerably closer to an ideal one-step, four-electron process (*n*=4.0). For the tolerance against CO poisoning (Supplementary Fig. 19a–c) and methanol crossover (Supplementary Fig. 19d), SbGnPs also displayed superior performance to the pristine graphite and Pt/C, suggesting that SbGnPs are one of the strongest alternative candidates for ORR electrocatalysts.

### Theoretical calculations

To investigate the origin of the high electrocatalytic activity and unusual cycle stability, we performed density functional theory (DFT) calculations (Supplementary Note 2). Based on the observations in the XPS spectra (see Fig. 3d), we calculated two distinct types of edge configurations for SbGnPs (Supplementary Fig. 13); namely, (i) bond formations between Sb atom and *sp*^*2*^ carbon atom at an armchair edge (Supplementary Fig. 13a,c), and (ii) the substitutions of Sb at the C site at a zigzag edge (Supplementary Fig. 13b.d). When the Sb atom formed a bond with C at the single *sp*^*2*^ C–Sb dangling bonds (Supplementary Fig. 12), the edge does not have particular binding affinity with the O_2_ molecule. The optimization through the total energy minimization revealed that the adsorption of O_2_ molecules is no more than intermolecular physisorption. However, when Sb atoms formed bonds with two C atoms in the armchair and zigzag edges as shown in Supplementary Fig. 13, the Sb edges revealed decent binding affinity with O_2_.

**Figure 5:**
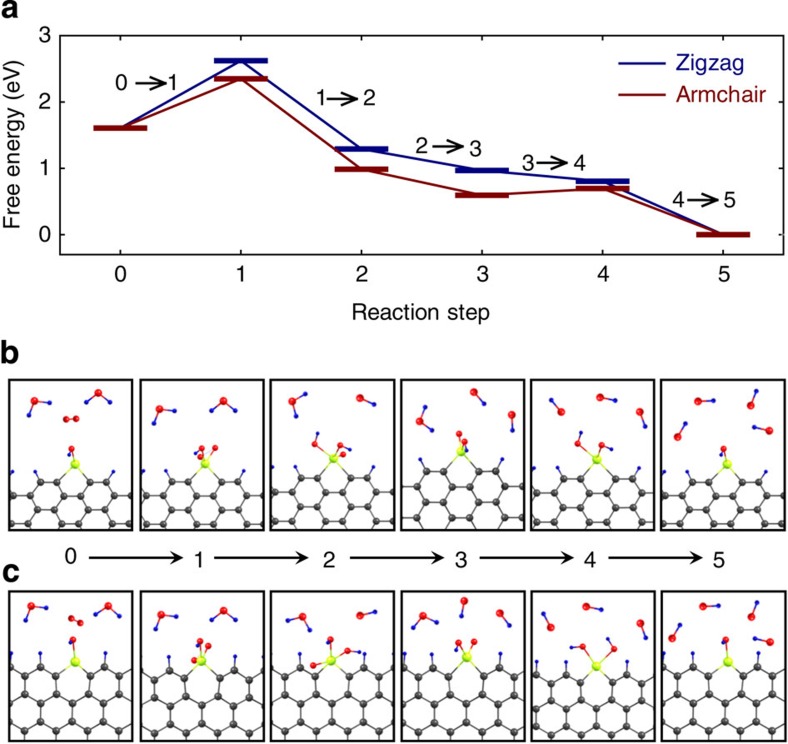
Oxygen reduction path I. (**a**) Free energy diagram along the ORR reaction steps on the armchair and zigzag edges of SbGnPs; (**b**) Atomic configurations at each intermediate step at armchair edges of SbGnPs; (**c**) Atomic configurations at each intermediate step at zigzag edges of SbGnPs.

Our DFT calculation results show that, besides the energy cost in the initial adsorption process of O_2_, the free energy gradually and monotonically decreased along the suggested reaction path with incoming water molecules (Fig. 5 and Table 1). The initial energy cost in this case is similar as that of the most well-known Pt catalyst^50^. The zigzag- and armchair-edged SbGnPs showed similar trend in the free energy profile (Fig. 5). Various other paths for the ORR are presented in Supplementary Fig. 20. It is important to notice that the calculated intermediate structures, during the ORR, oscillate between Sb^3+^ and Sb^5+^ (Fig. 5b,c and Supplementary Fig. 12). Assuming that the cycle instability is mainly related to the formation of hydrogen peroxide (H_2_O_2_), we compared the energetics of associative and dissociative paths. The total energy results at intermediate steps consistently indicate that the O_2_ is readily dissociated, and that the associative paths are unfavourable (Supplementary Fig. 21). This suggests that the formation of hydrogen peroxide (H_2_O_2_) is largely suppressed during the ORR process, supporting the observed cycle stability. The rotating ring disk electrode measurements also revealed consistent results. As presented in Supplementary Fig. 22, the use of SbGnPs suppressed the evolution of hydrogen peroxide even at the level of carbon-supported Pt electrode (Pt/C).

## Discussion

To the best of our knowledge, the present work is the first synthesis of SbGnPs via a simple mechanochemical ball-milling in solid state. The unique electrochemical performance of the SbGnPs suggests that they can be one of the best Pt alterative electrocatalysts, for which their precedents have inherent limitations. The comparison between the SbGnPs and other representative materials are shown in Supplementary Fig. 23 and their characteristics are summarized in Supplementary Table 5. For example, SbGnPs are the most cost-effective and the stable material for practical application (Supplementary Fig. 23).

In summary, we demonstrate, for the first time, Sb-doped GnPs using a green mechanochemical approach with simple ball-milling of graphite in the presence of solid Sb. The doping of Sb at the edges of GnPs was confirmed using various analytical techniques, including AR-TEM, HR-TEM, elemental analyses, XPS, TGA, EDS, SEM, X-ray diffraction, Raman, BET and TOF-SIMS. The results suggested that high-speed ball-milling can produce a sufficient amount of kinetic energy to generate active carbon and Sb species for the formation of graphitic C–Sb bonds. The resultant SbGnPs exhibited profoundly enhanced electrocatalytic performance for the cathodic ORR with tolerance against CO poisoning and methanol crossover. More importantly, SbGnPs displayed superb long-term stability compared with the pristine graphite and commercial Pt/C electrocatalysts. Furthermore, first-principle DFT calculations were performed for different edge configurations to understand the observed efficiency of the ORR and electrochemical stability. The free energy profile is favourable along the ORR paths through intermediates that alternate between the multiple oxidation states of Sb (Sb^3+^ and Sb^5+^). Furthermore, the dissociative processes are preferred to the associative ones, and thus the formation of hydrogen peroxide is suppressed. Our results suggest new insights and practical methods for designing edge-functionalized GnPs as high-performance metal-free ORR electrocatalysts using the low-cost and scalable ball-milling technique.

## Methods

### Syntheses of SbGnPs

SbGnPs were prepared simply by ball-milling graphite in the presence of Sb in a planetary ball-mill capsule. Graphite (5.0 g) and Sb (5.0 g) were placed into a stainless-steel capsule containing stainless-steel balls (500 g, diameter 5 mm). After the capsule was sealed, five cycles of charging (70 psi) of argon and discharging at reduced pressure (0.05 mm Hg) were applied to completely remove air. The capsule was then fixed in the planetary ball-mill machine, and agitated with 500 r.p.m. for 48 h. The resultant product was repetitively washed off to get rid of remaining metallic impurities and Sb with a warm concentrated HCl (∼37%), if any. Final product was Soxhlet extracted with concentrated HCl and then freeze-dried at −120 °C under a reduced pressure (0.05 mm Hg) for 48 h to yield dark black SbGnPs powder.

### Electrochemical measurements

The electrochemical measurements were conducted using a computer-controlled potentiostat (1470E Cell Test System, Solartron Analytical, UK) with a typical three-electrode cell. A platinum wire was used as a counter electrode and an Ag/AgCl (saturated KCl) electrode as the reference electrode. All the experiments were conducted at ambient condition. The working electrodes were prepared by applying respective catalyst inks onto the pre-polished glassy carbon disk electrodes. Briefly, samples were dispersed in N,N-dimethylformamide (DMF) and ultrasonicated for 5 min to form uniform catalyst inks (2 mg ml^−1^). A total of 10 μl of a well-dispersed catalyst ink was applied onto a pre-polished glassy carbon disk electrode (5 mm in diameter). After drying at room temperature, Nafion (0.05 wt.%) stock solution (1 μl) in ethanol was applied onto the surface of the catalyst layer to form a thin protective film.

The cyclic voltammograms were obtained in N_2_- and O_2_-saturated 0.1 M aq. KOH solution for ORR with various scan rates in the potential range from −0.9 to 0.1 V at room temperature. Rotating disk electrode measurements were performed in O_2_-saturated 0.1 M aq. KOH solution at rotation speed varying from 600 to 2,500 r.p.m. and with the scan rate of 10 mV s^−1^ from 0.1 to −0.9 V. The chronoamperometric response was tested at the polarizing potential of −0.3 V at 2,500 r.p.m. in O_2_-saturated electrolyte by bubbling O_2_ into the electrolyte. During this process, 3 M methanol (2.0 ml) or flow of CO and nitrogen gas was introduced into the electrolyte at 400 s to examine the methanol crossover and CO poisoning effect, respectively.

The detailed kinetic analysis was conducted according to Koutecky–Levich plots in equation (1):


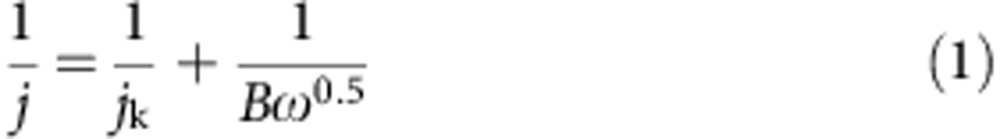


where *j*_k_ is the kinetic current and *B* is Levich slope which is given by equation (2):


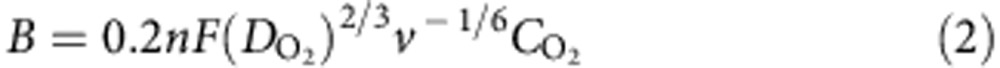


Here *n* is the number of electrons transferred in the reduction of one O_2_ molecule, *F* is the Faraday constant (*F*=96485 C mol^−1^), *D*_O2_ is the diffusion coefficient of O_2_ (*D*_O2_=1.9 × 10^−5^ cm^2^ s^−1^), *ν* is the kinematics viscosity for KOH (*ν*=0.01 cm^2^ s^−1^) and *C*_O2_ is concentration of O_2_ in the solution (*C*_O2_=1.2 × 10^−6^ mol cm^−3^). The constant 0.2 is adopted when the rotation speed is expressed in r.p.m. According to equations (1) and (2), the number of electrons transferred (*n*) can be obtained from the slope of Koutecky–Levich plot of *i*^−1^ versus *ω*^−1/2^. From published data for *F* (96,485 C mol^−1^), *D*_O2_ (1.9 × 10^−5^ cm^2^ s^−1^), *ν* (0.01 cm^2^ s^−1^) and *C*_O2_ (1.2 × 10^−6 ^mol cm^−3^), *B* is calculated to be 0.144 mA s^−1/2^ at *A*=0.19625, cm^2^ for a four-electron exchange reaction (*n*=4).

## Additional information

**How to cite this article:** Jeon, I.-Y. *et al*. Antimony-doped graphene nanoplatelets. *Nat. Commun.* 6:7123 doi: 10.1038/ncomms8123 (2015).

## Supplementary Material

Supplementary Figures, Supplementary Tables, Supplementary Notes, Supplementary Methods and Supplementary References. Supplementary Figures 1-23, Supplementary Tables 1-5, Supplementary Notes 1-2, Supplementary Methods and Supplementary References

Supplementary Movie 1 *In-situ* TEM observation showing the stability of SbGnPs under e-beam irradiation.

Supplementary Movie 2 *In-situ* TEM observation showing Cu-mediated etching of CVD graphene under e-beam irradiation.

## Figures and Tables

**Table 1:** Detailed reaction mechanisms at each intermediate step of oxygen reduction path I described in Fig. 5.

**Reaction step**	**Reaction path**
0→1	Sb-OH+O_2_+2H_2_O+4e^−^→Sb-OH(O_2_)+2H_2_O+4e^−^
1→2	Sb-OH(O_2_)+2H_2_O+4e^−^→Sb-OH(OOH)+OH^−^+H_2_O+3e^−^
2→3	Sb-OH(OOH)+OH^−^+H_2_O+3e^−^→Sb-OH(O)+2OH^−^+H_2_O+2e^−^
3→4	Sb-OH(O)+2OH^−^+H_2_O+2e^−^→Sb-OH(OH)+3OH^−^+e^−^
4→5	Sb-OH(OH)+3OH^−^+e^−^→Sb-OH+4OH^−^
