# Supplementary material for: Antimony-doped graphene nanoplatelets
Source: Nat Commun. 2015 May 22;6:7123. doi: 10.1038/ncomms8123 (PMC4455135; doi:10.1038/ncomms8123)
Supplement: Supplementary Figures, Supplementary Tables, Supplementary Notes, Supplementary Methods and Supplementary References. — Supplementary Figures 1-23, Supplementary Tables 1-5, Supplementary Notes 1-2, Supplementary Methods and Supplementary References [file ncomms8123-s1.pdf]

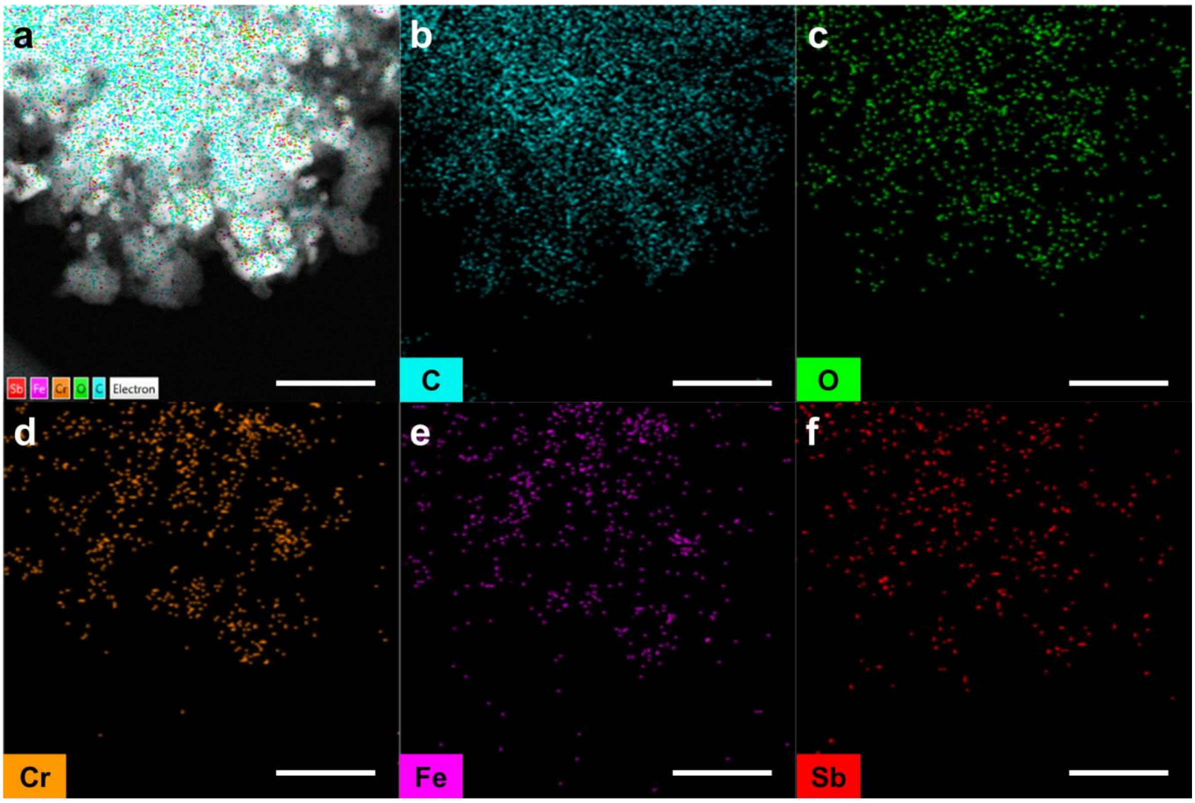

Supplementary Figure 1 | (a) High-angle annular dark field (HAADF) scanning TEM (STEM) image of SbGnPs after washing with warm concentrated hydrochloric acid (~37 %) as described in Supplementary Methods (before Soxhlet extraction with concentrated hydrochloric acid). Corresponding element mappings: **(b)** Carbon; **(c)** Oxygen; **(d)** Chromium; **(c)** Iron; **(f)** Antimony. Scale bars are 20 nm.

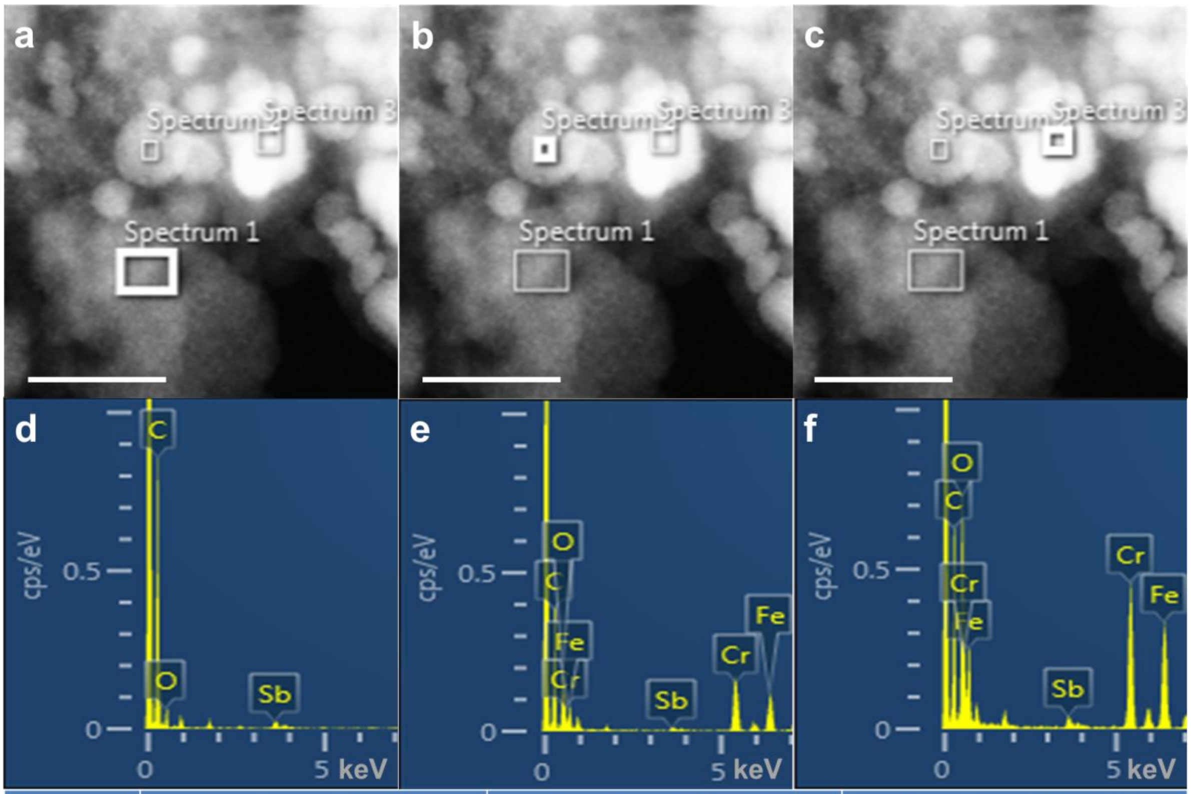

Element	Spectrum 1 (at.%)	Spectrum 2 (at.%)	Spectrum 3 (at.%)
C	90.43	51.54	34.08
O	5.04	15.36	25.34
Cr	BD	17.91	20.63
Fe	BD	13.34	16.73
Sb	4.53	1.85	3.23

Supplementary Figure 2 | (a, b, c) High-angle annular dark field (HAADF) scanning TEM (STEM) image of SbGnPs before complete work-up procedure (before Soxhlet extraction with concentrated hydrochloric acid) as described in Supplementary Methods. Scale bars are 20 nm. **(d, e, f)** Energy-dispersive X-ray (EDX) spectra of SbGnPs. The table shows corresponding element contents (atomic percentage), indicating that bright particles are metal debris.

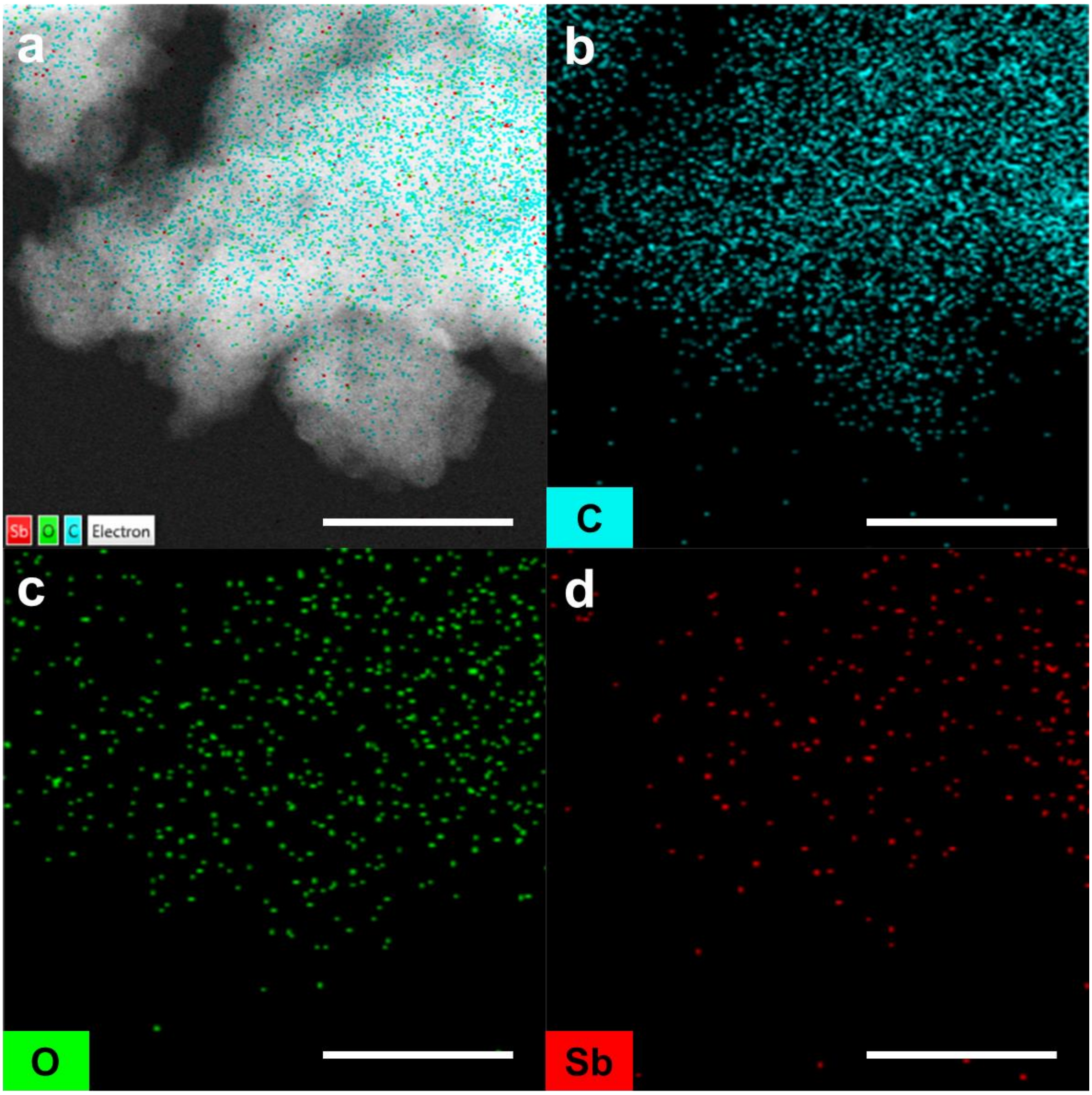

Supplementary Figure 3 | (a) High-angle annular dark field (HAADF) scanning TEM (STEM) image of SbGnPs after complete work-up procedure (after Soxhlet extraction with concentrated hydrochloric acid) as described in Supplementary Methods. Corresponding element mappings: **(b)** Carbon; **(c)** Oxygen; **(d)** Antimony. Scale bars are 20 nm.

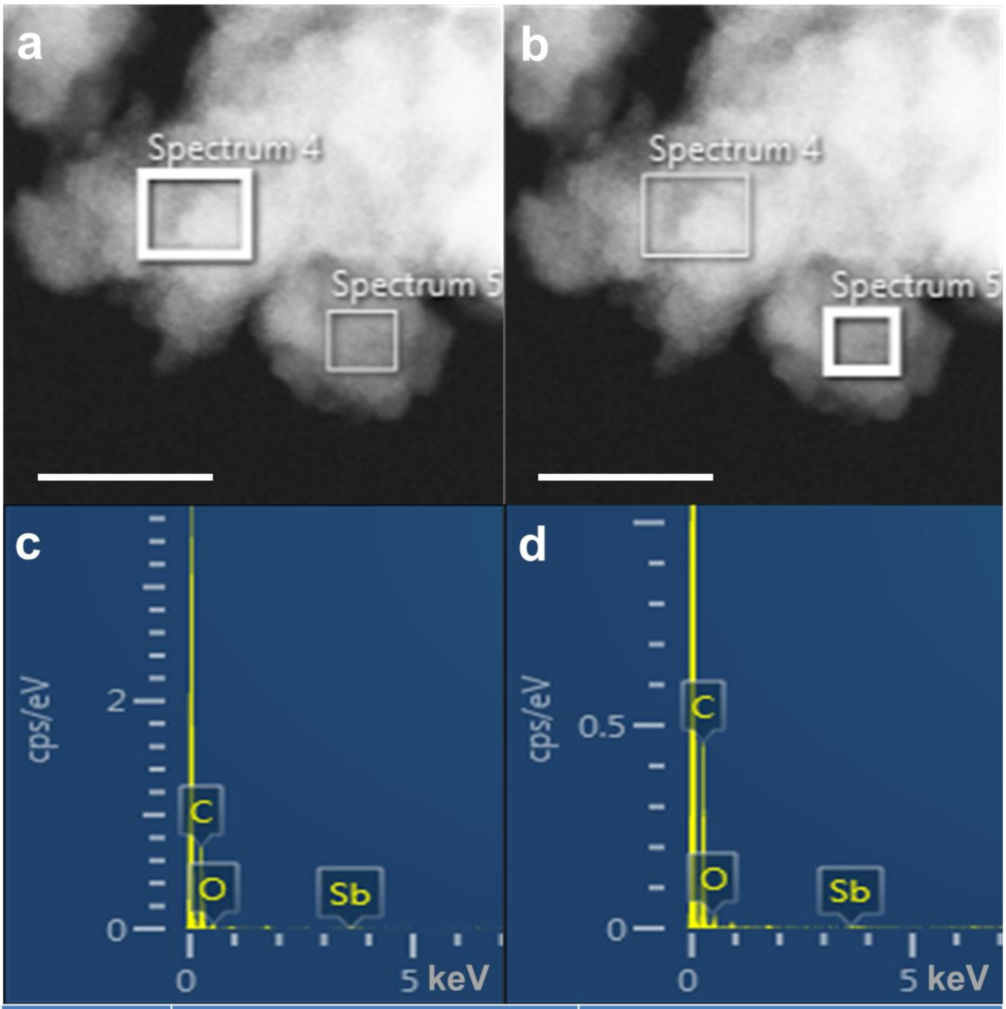

Element	Spectrum 4 (at.%)	Spectrum 5 (at.%)
C	93.63	95.00
O	3.20	4.40
Cr	BD	BD
Fe	BD	BD
Sb	3.17	0.60

Supplementary Figure 4 | (a, b) High-angle annular dark field (HAADF) scanning TEM (STEM) image of SbGnPs after complete work-up procedure (after Soxhlet extraction with concentrated hydrochloric acid) as described in Supplementary Methods. Scale bars are 20 nm. **(c, d)** Energy-dispersive X-ray (EDX) spectra of SbGnPs. The table shows corresponding element contents (atomic percentage).

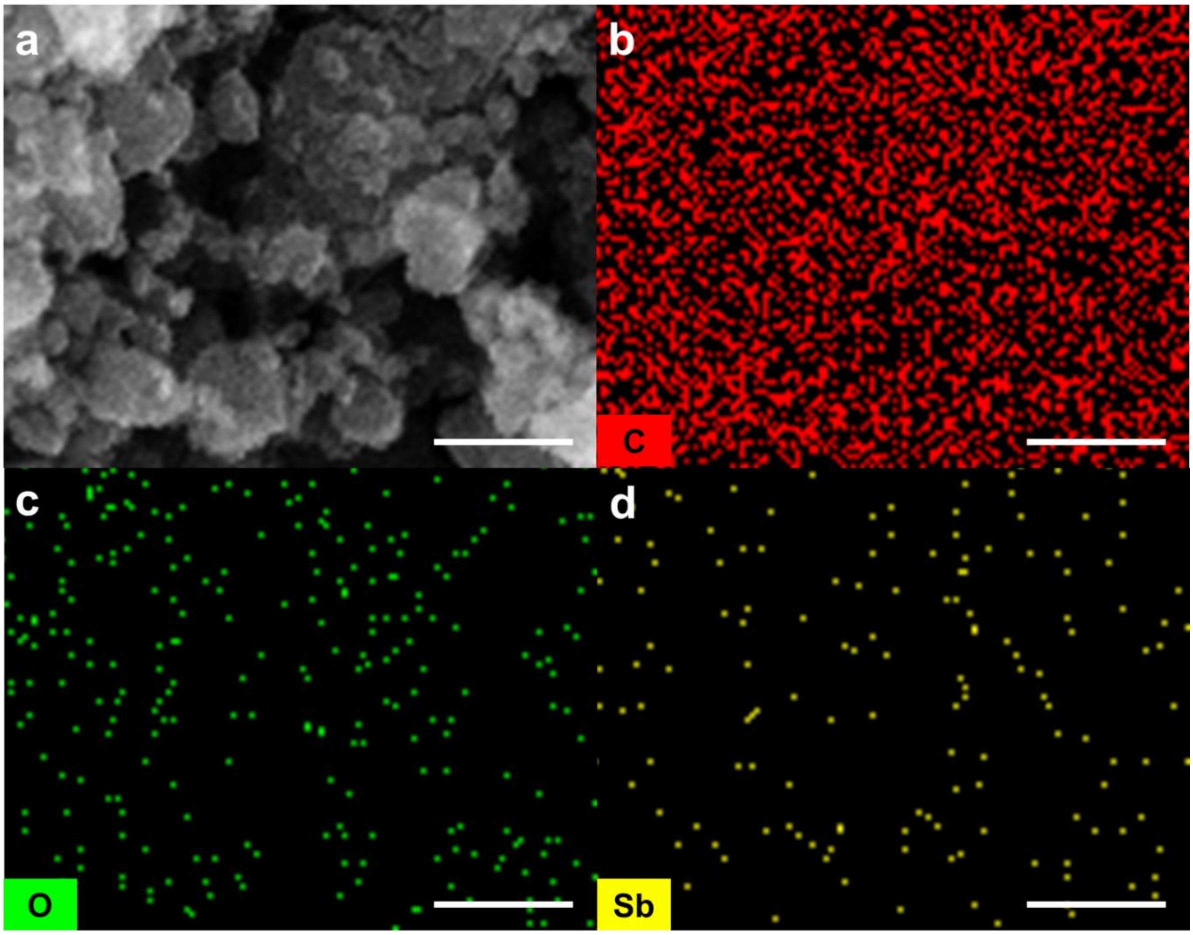

Supplementary Figure 5 | (a) FE-SEM image of SbGnPs after Soxhlet extraction with concentrated hydrochloric acid. Corresponding element mappings: **(b)** Carbon; **(c)** Oxygen; **(d)** Antimony. Scale bars are 250 nm.

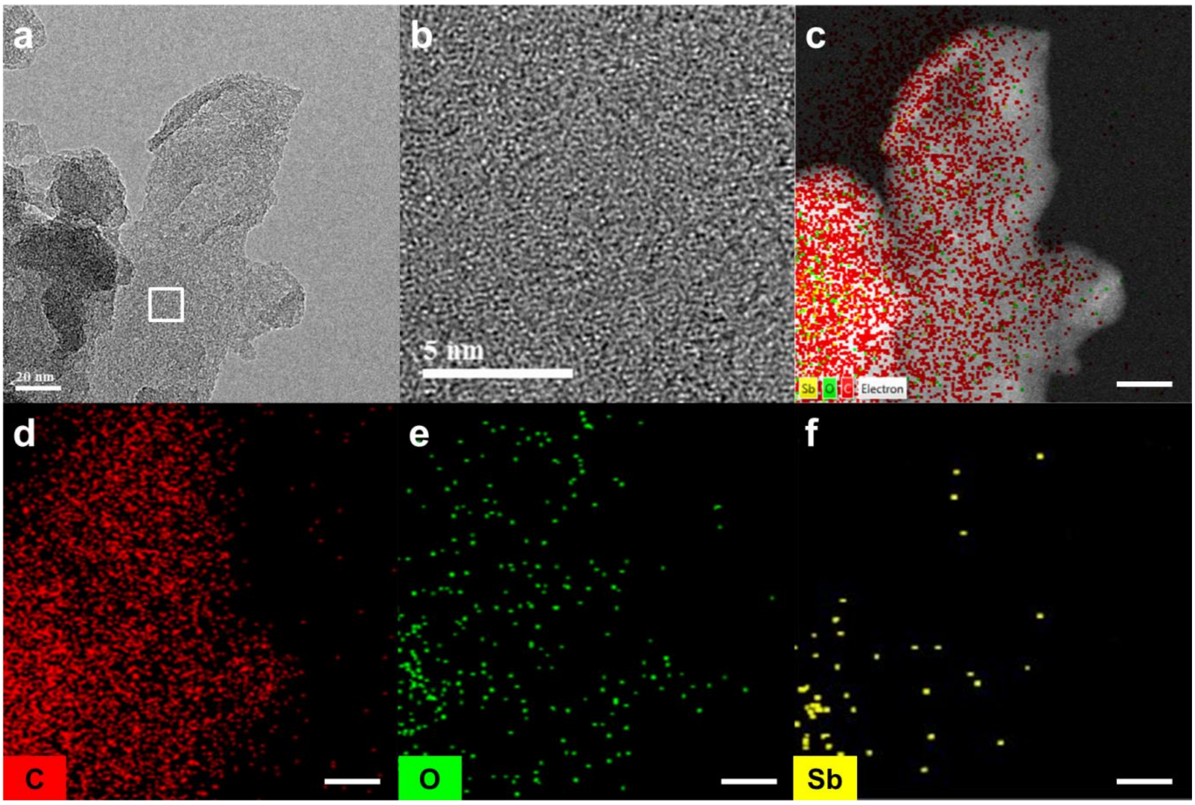

Supplementary Figure 6 | (a) High-angle annular dark field (HAADF) scanning TEM (STEM) image of SbGnPs after complete work-up procedure (after Soxhlet extraction with concentrated hydrochloric acid) as described in Supplementary Methods; **(b)** The magnified view of square in **a** at higher magnification, showing that graphitic lattice patterns indicate high crystallinity; **(c)** Dark-field (DF) TEM image of **a** with element mappings; **(d)** Carbon; **(e)** Oxygen; **(f)** Antimony. Scale bars are 20 nm.

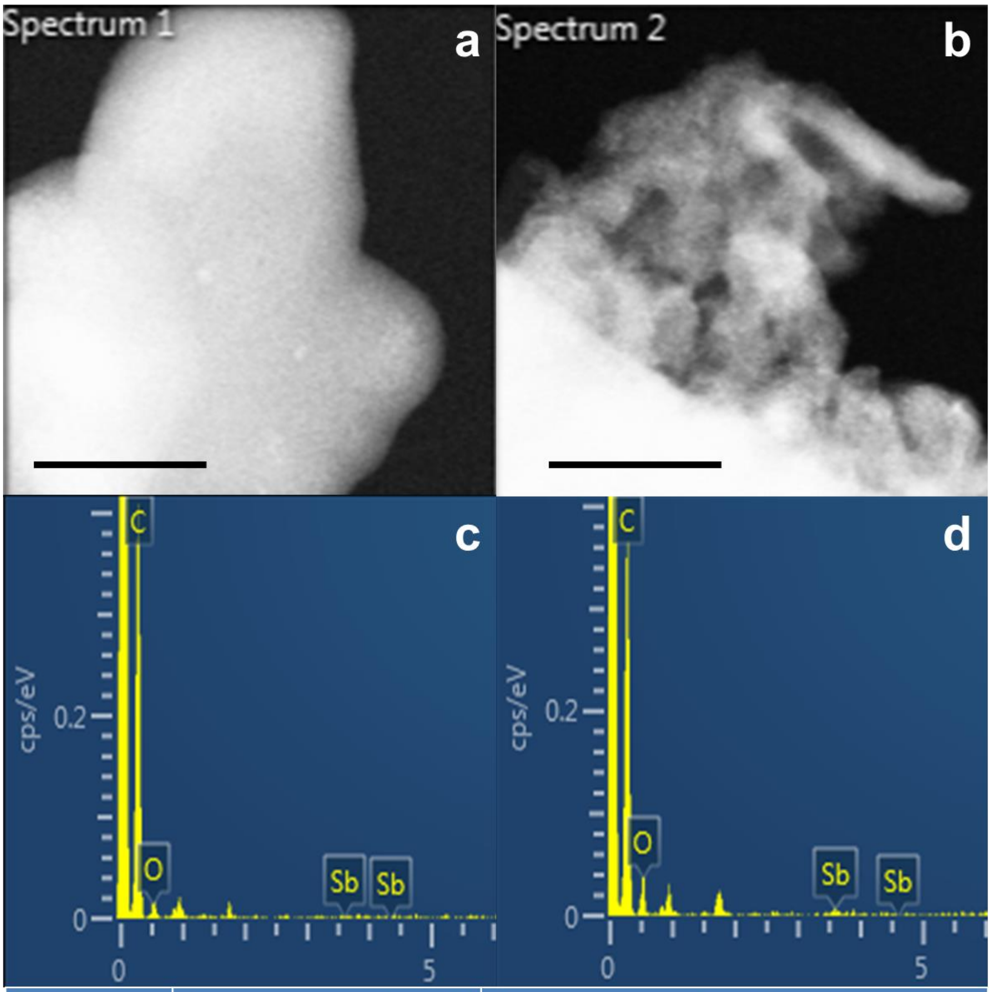

Element	Spectrum 1 (at.%)	Spectrum 2 (at.%)	Average
**C**	96.82	92.13	**94.48**
**O**	2.47	6.03	**4.25**
**Cr**	BD	BD	**BD**
**Fe**	BD	BD	**BD**
**Sb**	0.71	1.84	**1.28**

Supplementary Figure 7 | (a, b) Dark-field (DF) TEM image of SbGnPs after complete work-up procedure (after Soxhlet extraction with concentrated hydrochloric acid) as described in Supplementary Methods. Scale bars are 20 nm. **(c, d)** Energy-dispersive X-ray (EDX) spectra of SbGnPs. The table shows corresponding element contents (atomic percentage).

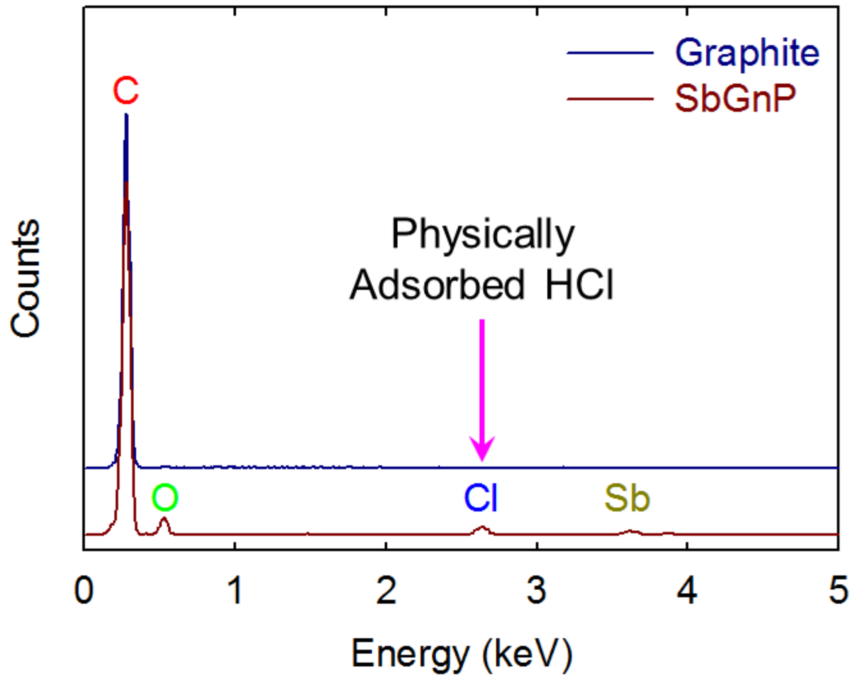

Supplementary Figure 8 | FE-SEM energy-dispersive X-ray (EDX) spectra, displaying Sb in addition to C, O and physically adsorbed Cl.

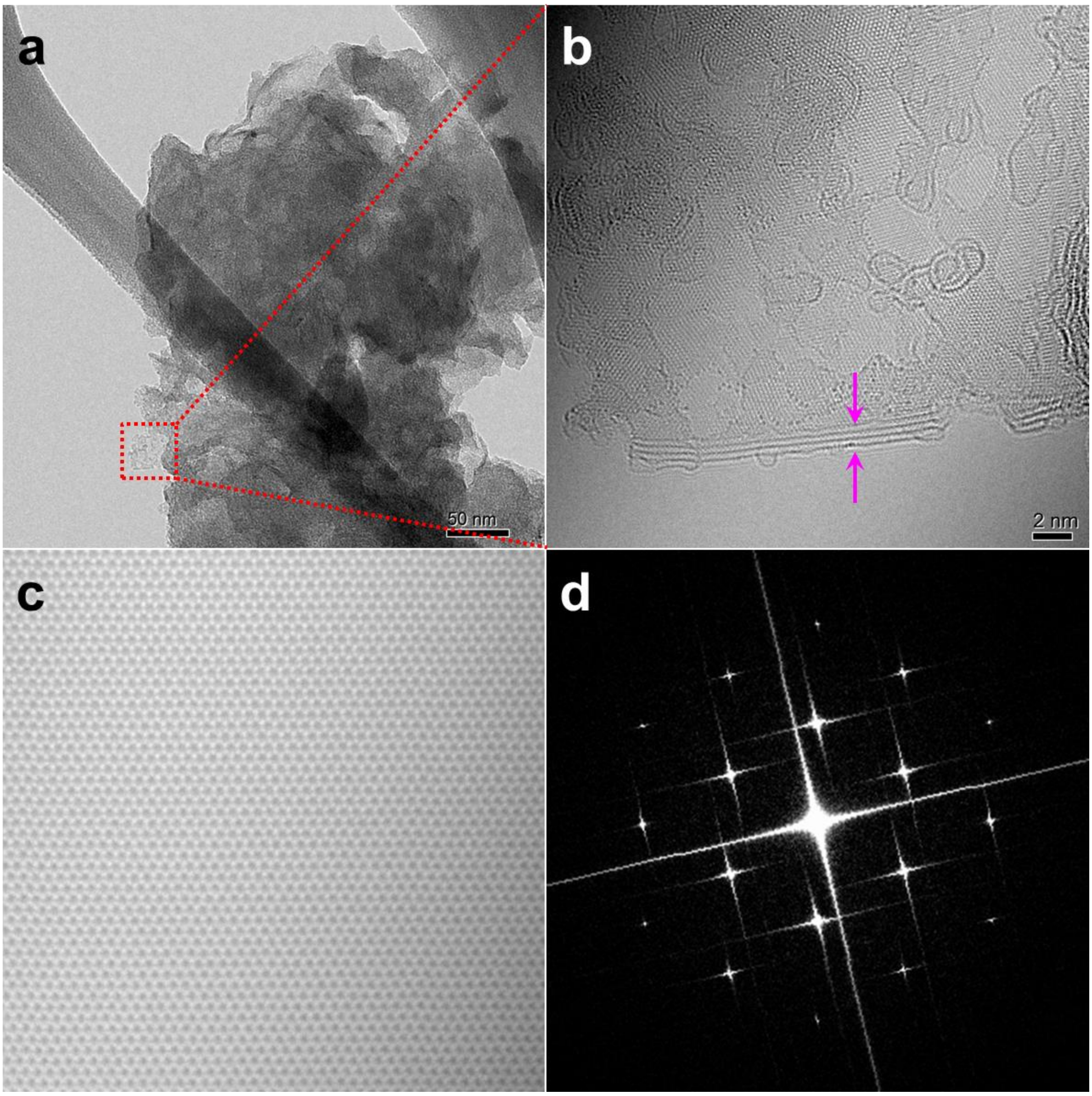

Supplementary Figure 9 | Atomic-resolution TEM (AR-TEM) images of SbGnPs and CVD grown graphene as reference: **(a)** Low-magnification TEM image of SbGnPs; **(b)** High-magnification of SbGnPs; **(c)** High-magnification TEM image of CVD graphene; **(d)** The fast Fourier transform (FFT) of the image in **(c)**.

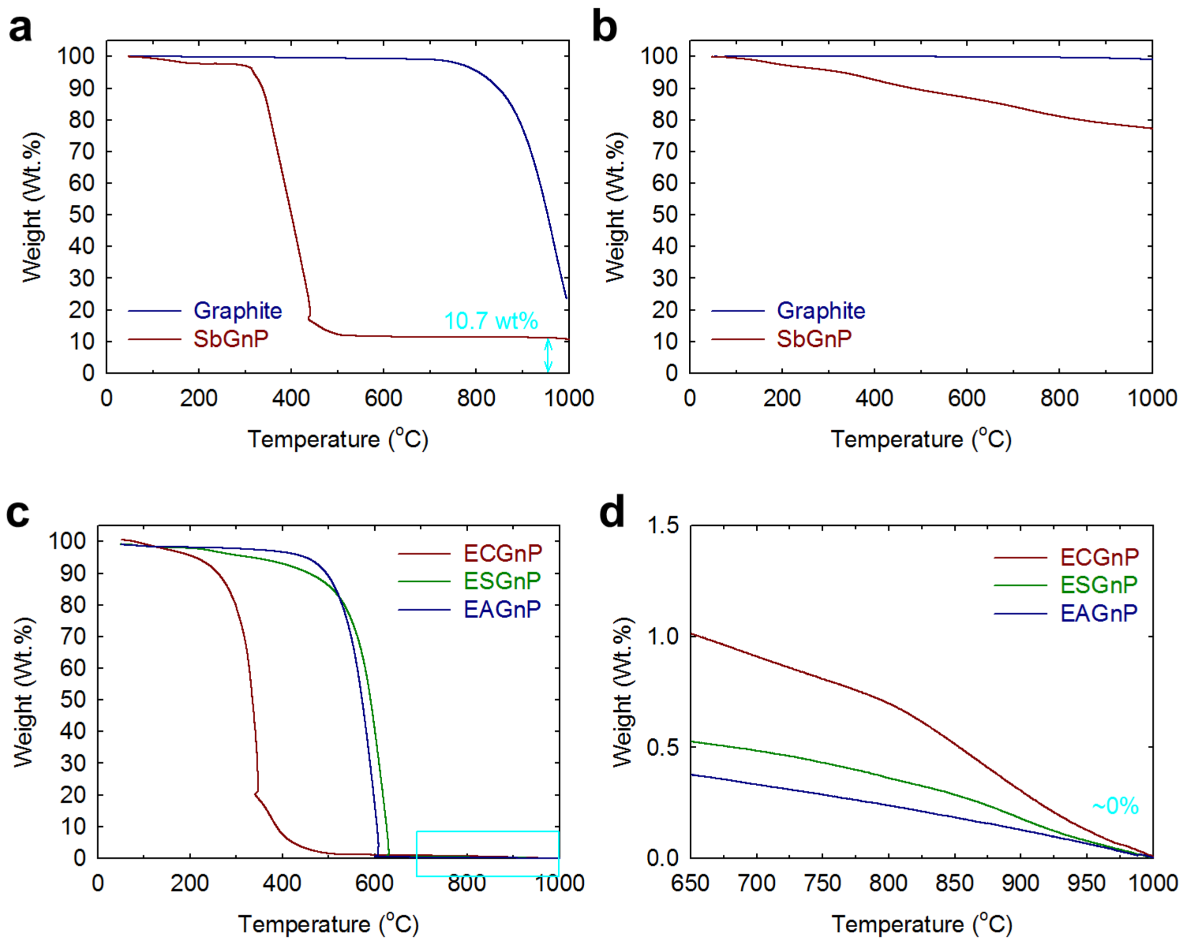

Supplementary Figure 10 | TGA thermograms of the pristine graphite and SbGnPs obtained at the heating rate of 10 °C min^{-1}: **(a)** In air; **(b)** In nitrogen. **(c)** TGA thermograms other EFGnPs (ECGnPs, ESGnPs and EAGnPs prepared by mechanochemical ball-milling for 48 hours in the presence of CO_2 (dry ice), SO_3, NH_3, respectively, obtained at the heating rate 10 °C min^{-1} in air; **(d)** High-resolution TGA thermograms at around 1,000 °C indicated that there are no metallic residues after complete work-up procedures.

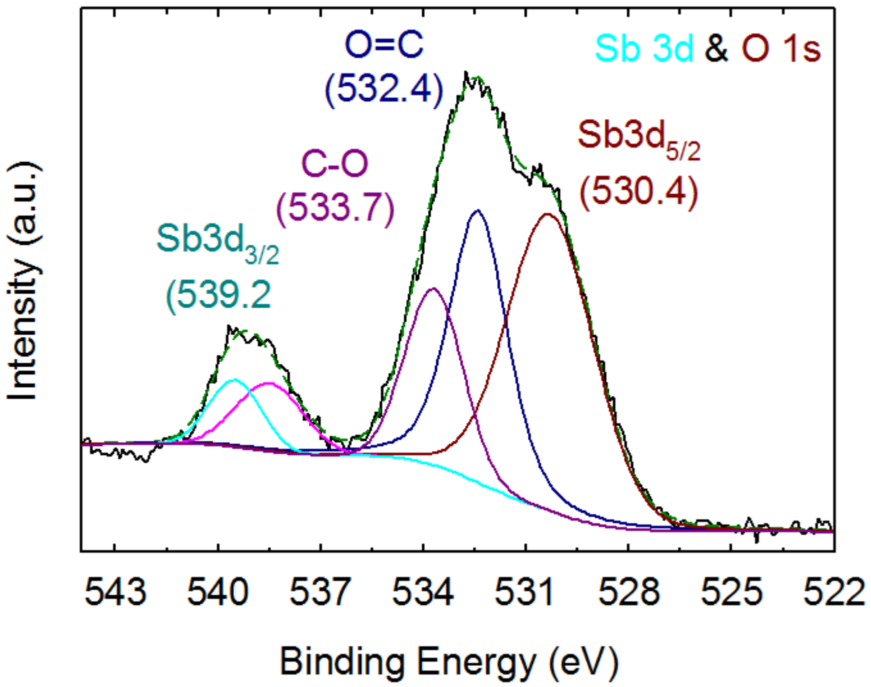

Supplementary Figure 11 | High-resolution XPS spectra with deconvolution for O1s and Sb3d. The ratio between Sb^{3+} and Sb^{5+} in $Sb3d_{3/2}$ is approximately 1, suggesting that the population of proposed structures Sb^{3+} and Sb^{5+} in Supplementary Fig. S12 should be similar.

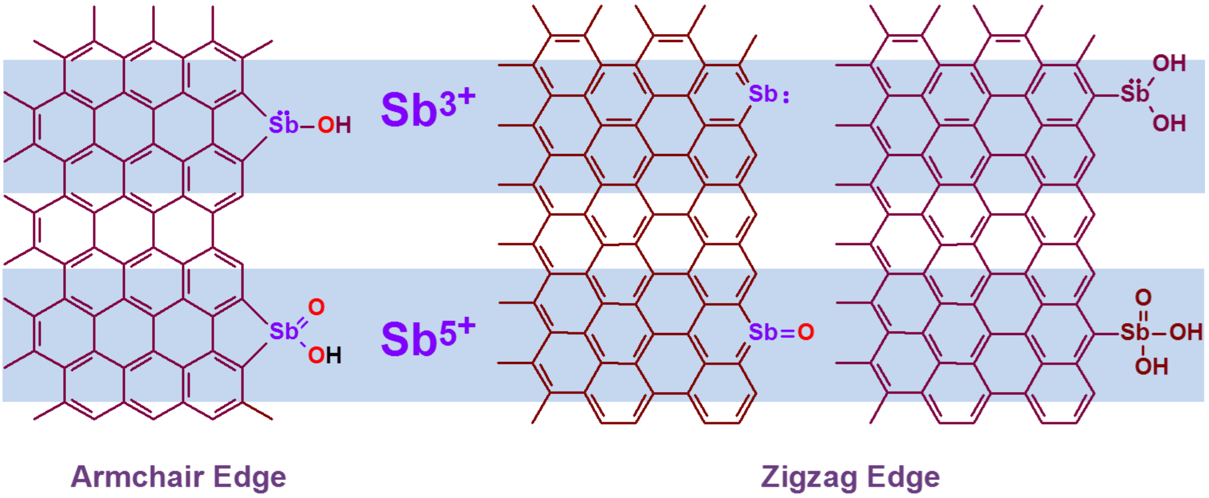

Supplementary Figure 12 | The proposed chemical natures of Sb on the armchair and zigzag edges of SbGnPs.

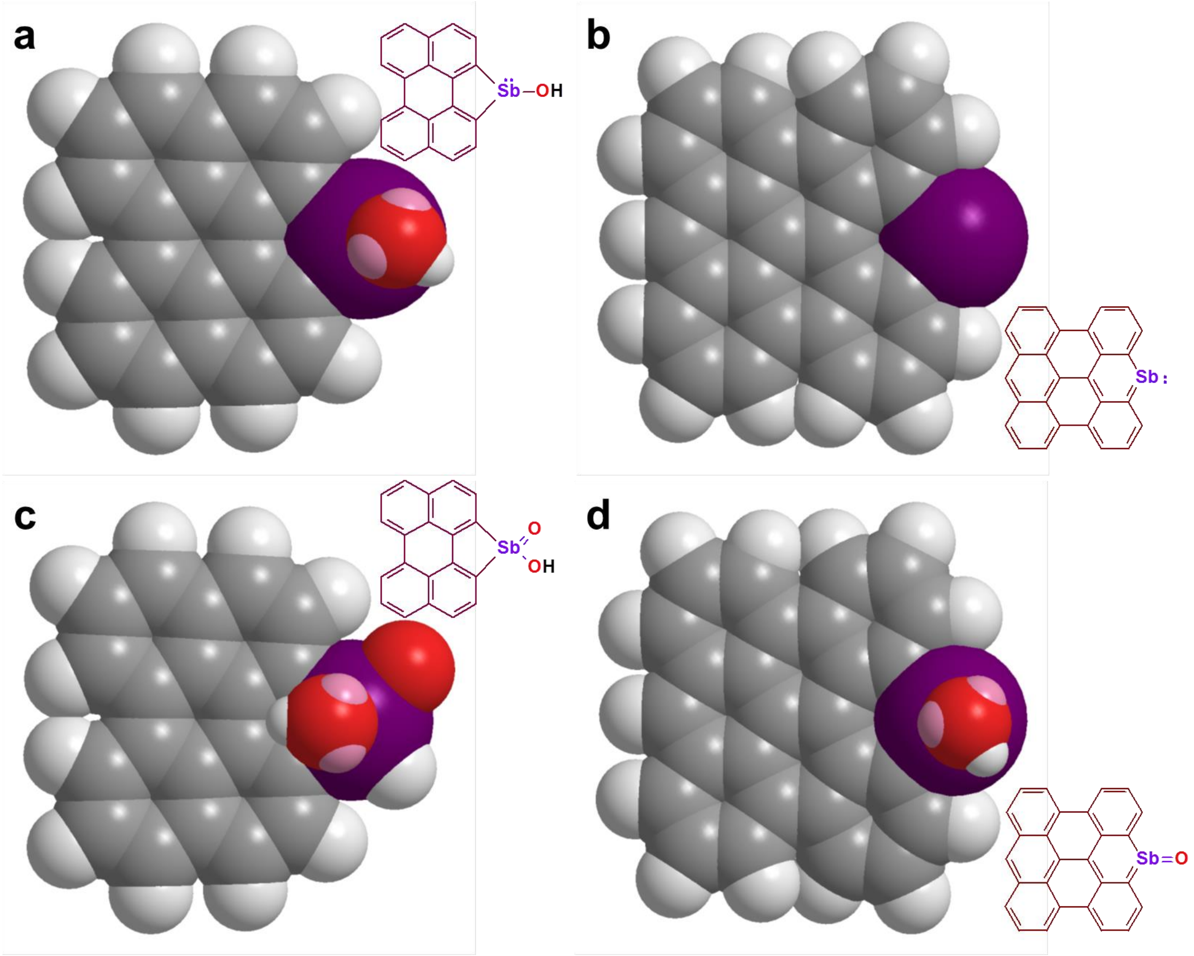

Supplementary Figure 13 | Four possible structures among the proposed edge-types of SbGnPs in Supplementary Fig. 12 after suffering the harsh work-up procedures: **(a)** Sb-OH; **(b)** Sb; **(c)** Sb(=O)OH; **(d)** Sb=O. Note that the space-filling molecular models depicts the edge shapes but the actual grain sizes of SbGnPs. The grain sizes of SbGnPs synthesized in the present work are much bigger than these models.

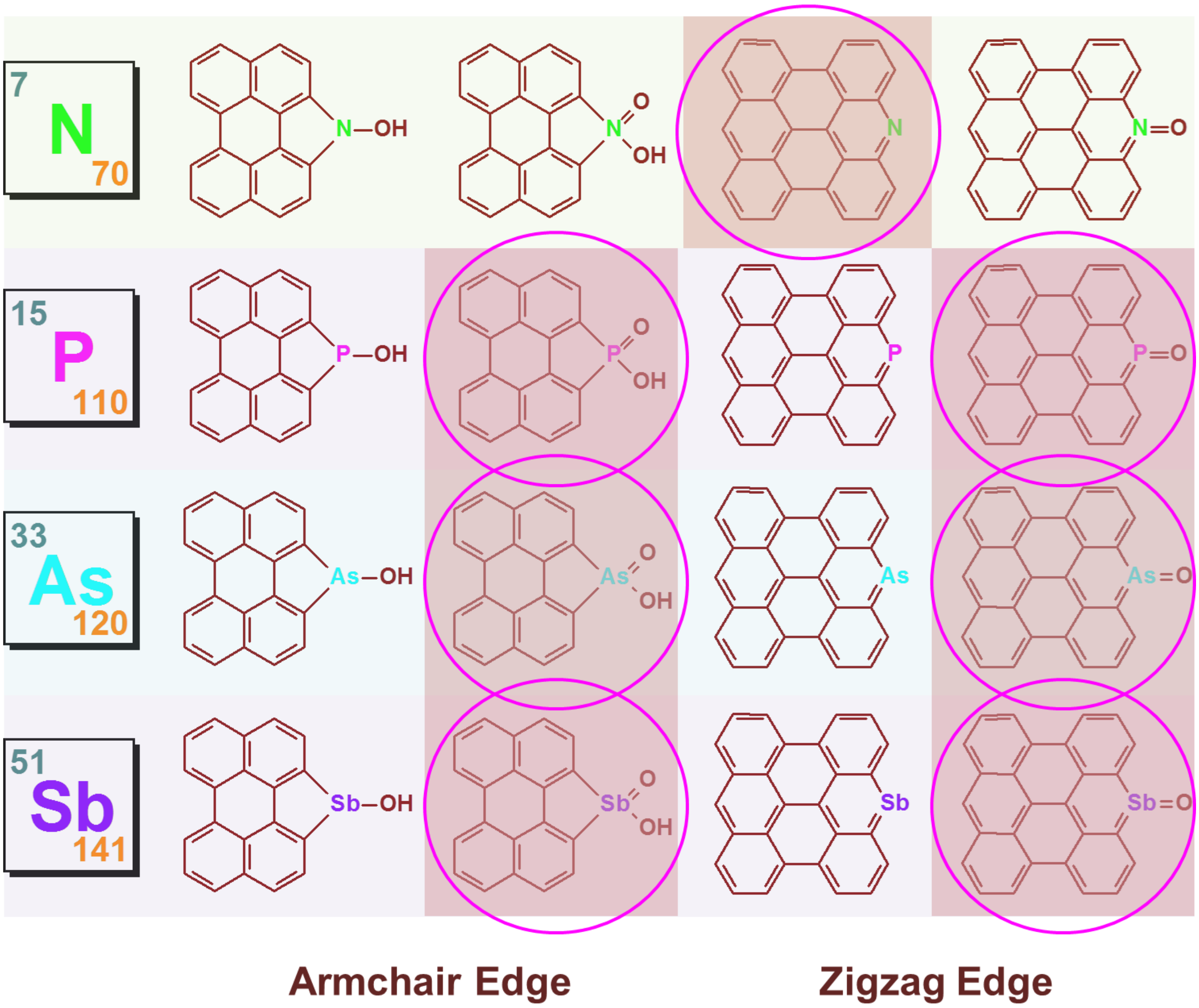

Supplementary Figure 14 | The rationalized edge-structures of GnPs with Group VA elements in periodic table. Pink circles are the proposed stable structures of each element.

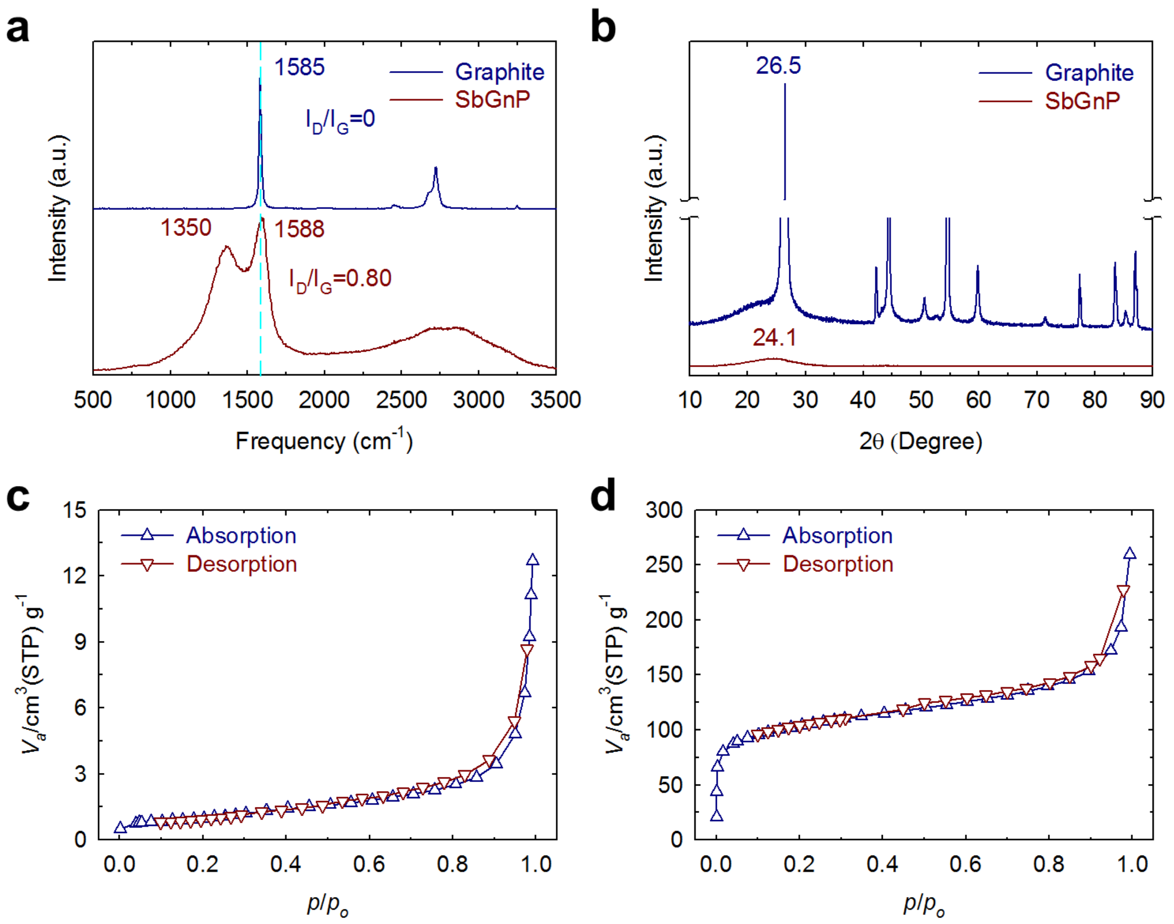

Supplementary Figure 15 | (a) Raman spectra; **(b)** XRD diffraction patterns. Nitrogen adsorption-desorption isotherms obtained at 77 K: **(c)** The pristine graphite; **(d)** SbGnPs.

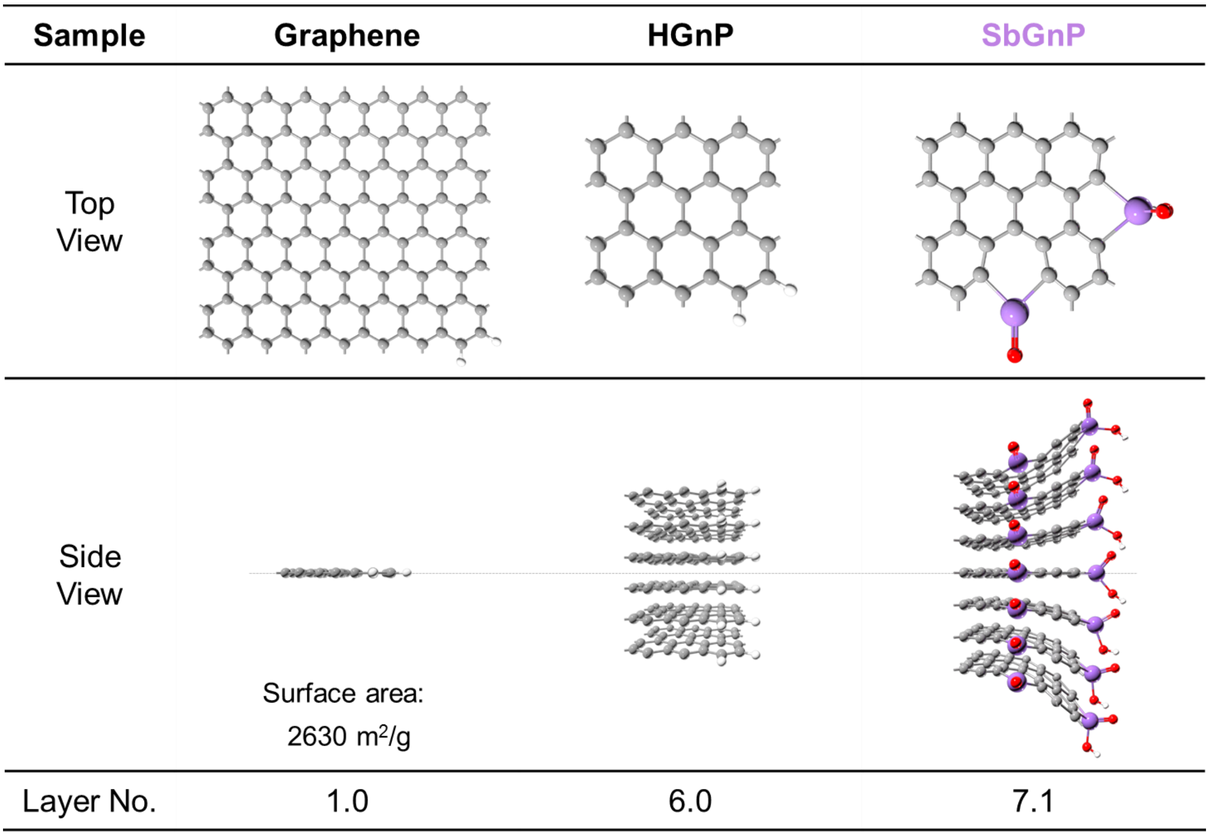

Supplementary Figure 16 | The top and side view images and calculated graphitic layer number of graphene nanoplatelets HGnPs (As a reference, HGnPs was prepared by ball-milling graphite in the presence of H_2) and SbGnPs. SbGnPs were effectively edge-delaminated through cracking graphitic layers and doping bulky Sb at the edges of SbGnPs.

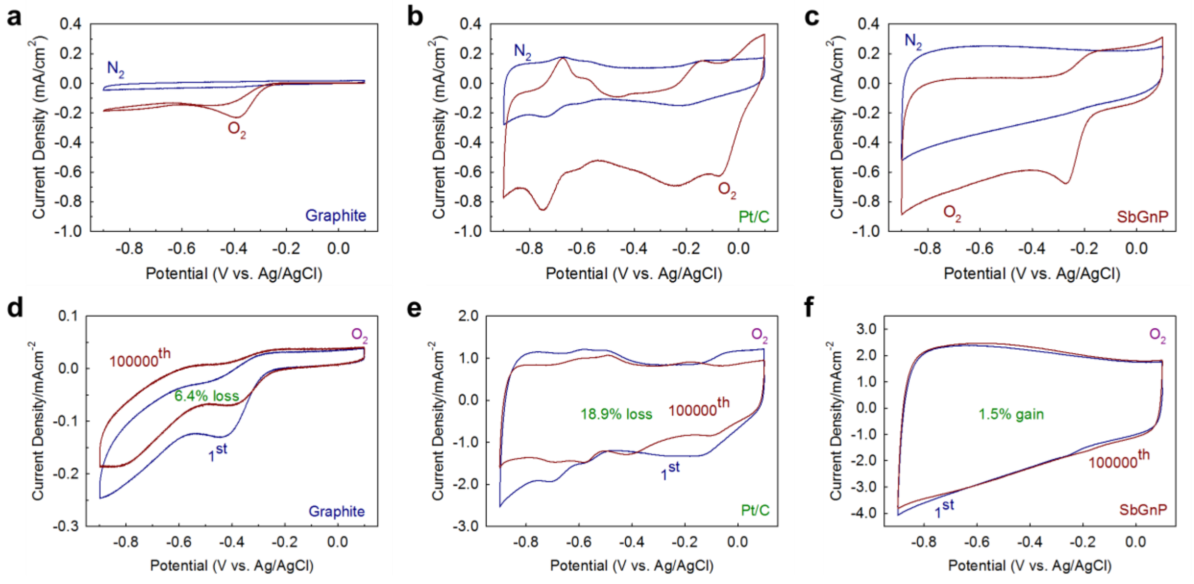

Supplementary Figure 17 | CV curves obtained from the sample electrodes in N_2- and O_2-saturated 0.1 M aq. KOH solutions at a scan rate of 10 mV s^{-1}: **(a)** The pristine graphite; **(b)** Pt/C; **(c)** SbGnPs. CV curves of 1st and $100,000^{th}$ cycles with scan rate of 100 mV s^{-1} in O_2-saturated 0.1 M aq. KOH solution: **(d)** The pristine graphite; **(e)** Pt/C; **(f)** SbGnPs.

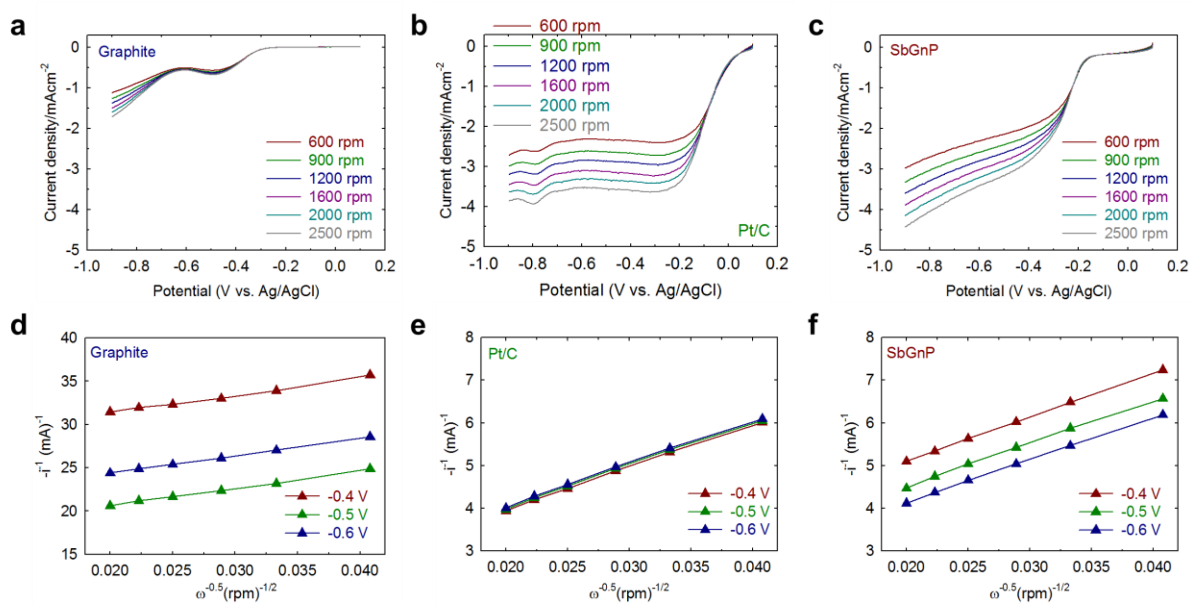

Supplementary Figure 18 | RDE voltammograms in O_2-saturated 0.1 M aq. KOH solution at a scan rate of 10 mV s^{-1} at different rotating rates of 600, 900, 1200, 1600, 2000 and 2500 rpm: **(a)** the pristine graphite; **(b)** Pt/C; **(c)** SbGnPs. Koutecky-Levich plots derived from the RDE measurements at different electrode potentials: **(d)** the pristine graphite; **(e)** Pt/C; **(f)** SbGnPs.

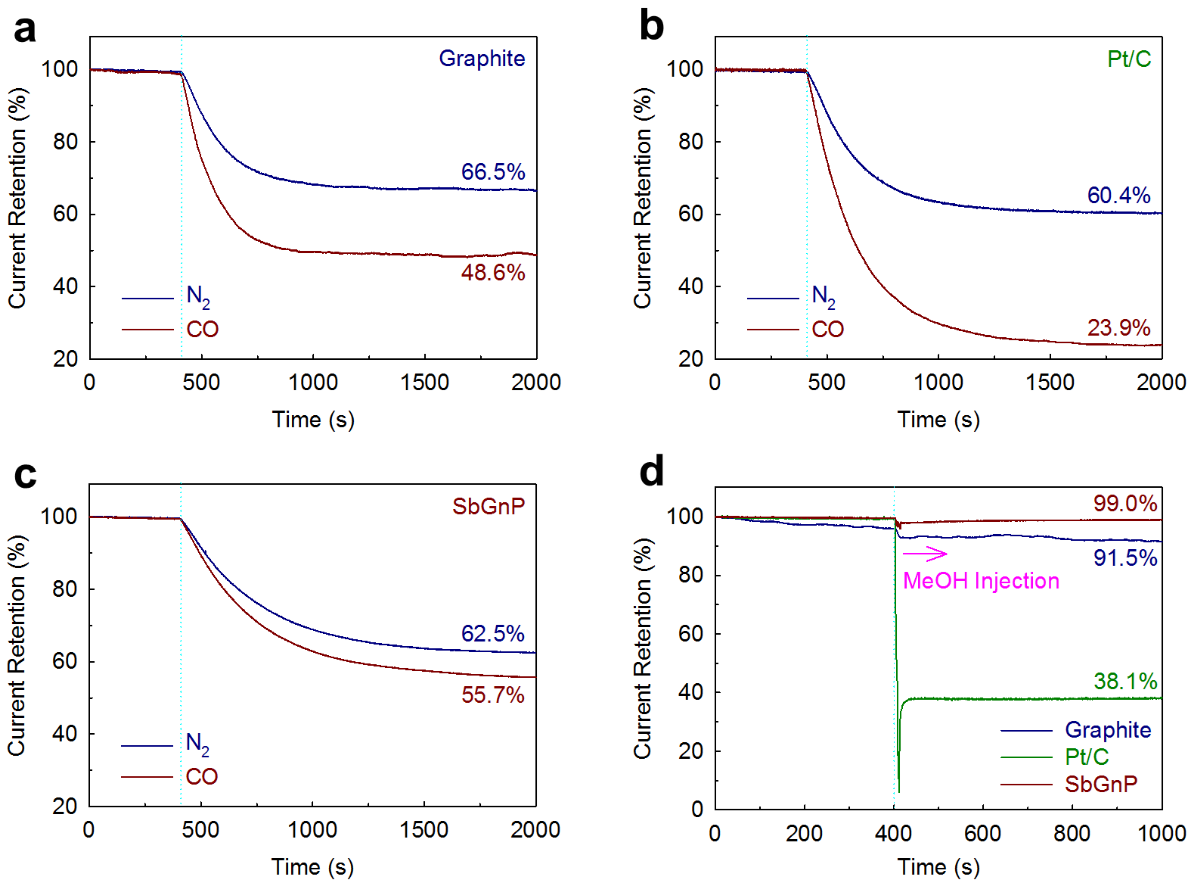

Supplementary Figure 19 | The current time (*j*-*t*) chronoamperometric response at -0.3 V (*vs.* Ag/AgCl) and rotation rate of 2500 rpm by CO and nitrogen gas injection: **(a)** The pristine graphite; **(b)** Pt/C; **(c)** SbGnPs. **(d)** The current time (*j*-*t*) chronoamperometric response at -0.3 V (*vs.* Ag/AgCl) and rotation rate of 2500 rpm by 3 M methanol (2 mL) injection.

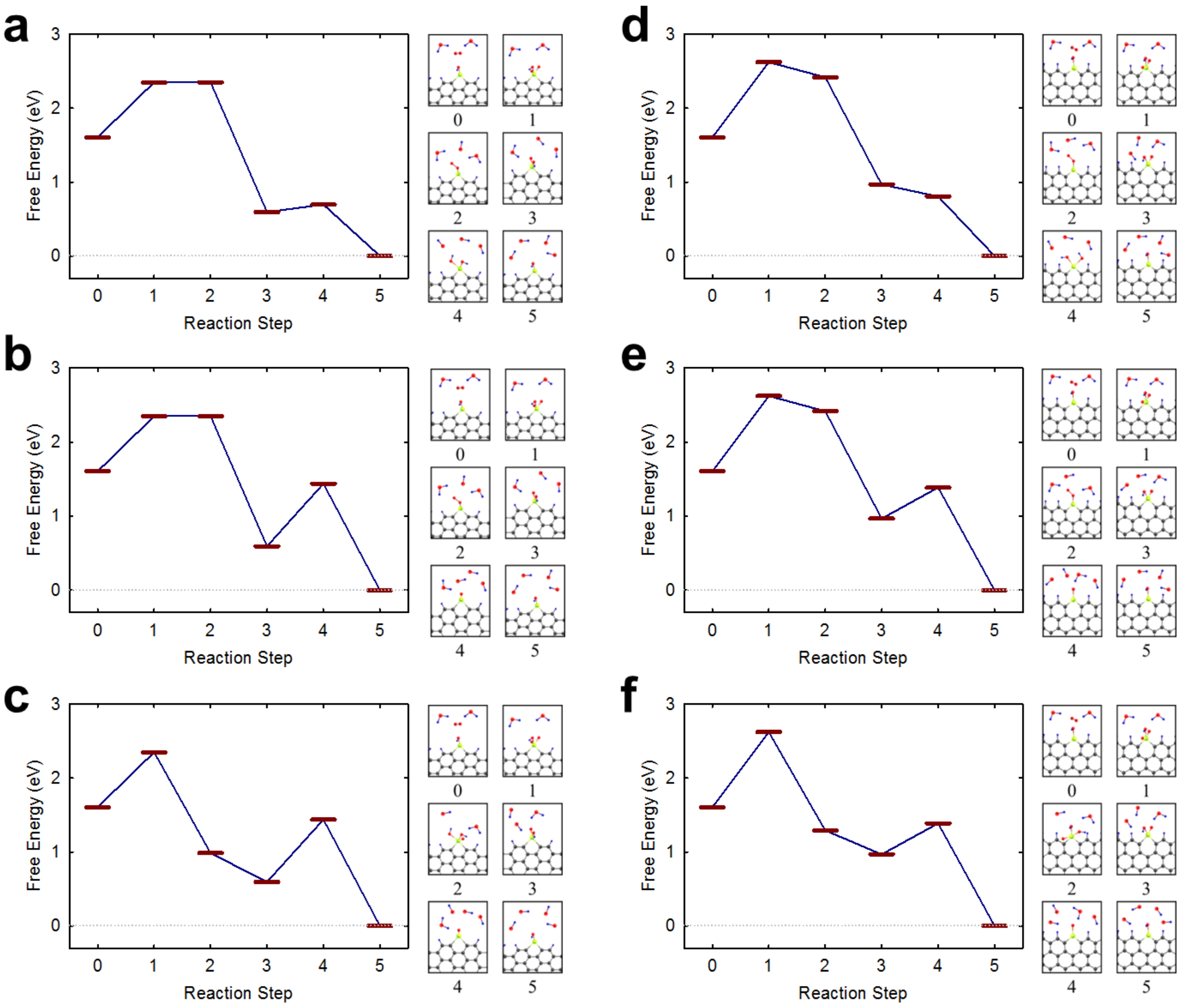

Supplementary Figure 20 | ORR energy profile along various other reaction paths: (**a-c**) on Sb-adsorbed armchair edges of SbGnPs; (**d-f**) on Sb-adsorbed zigzag edges of SbGnPs.

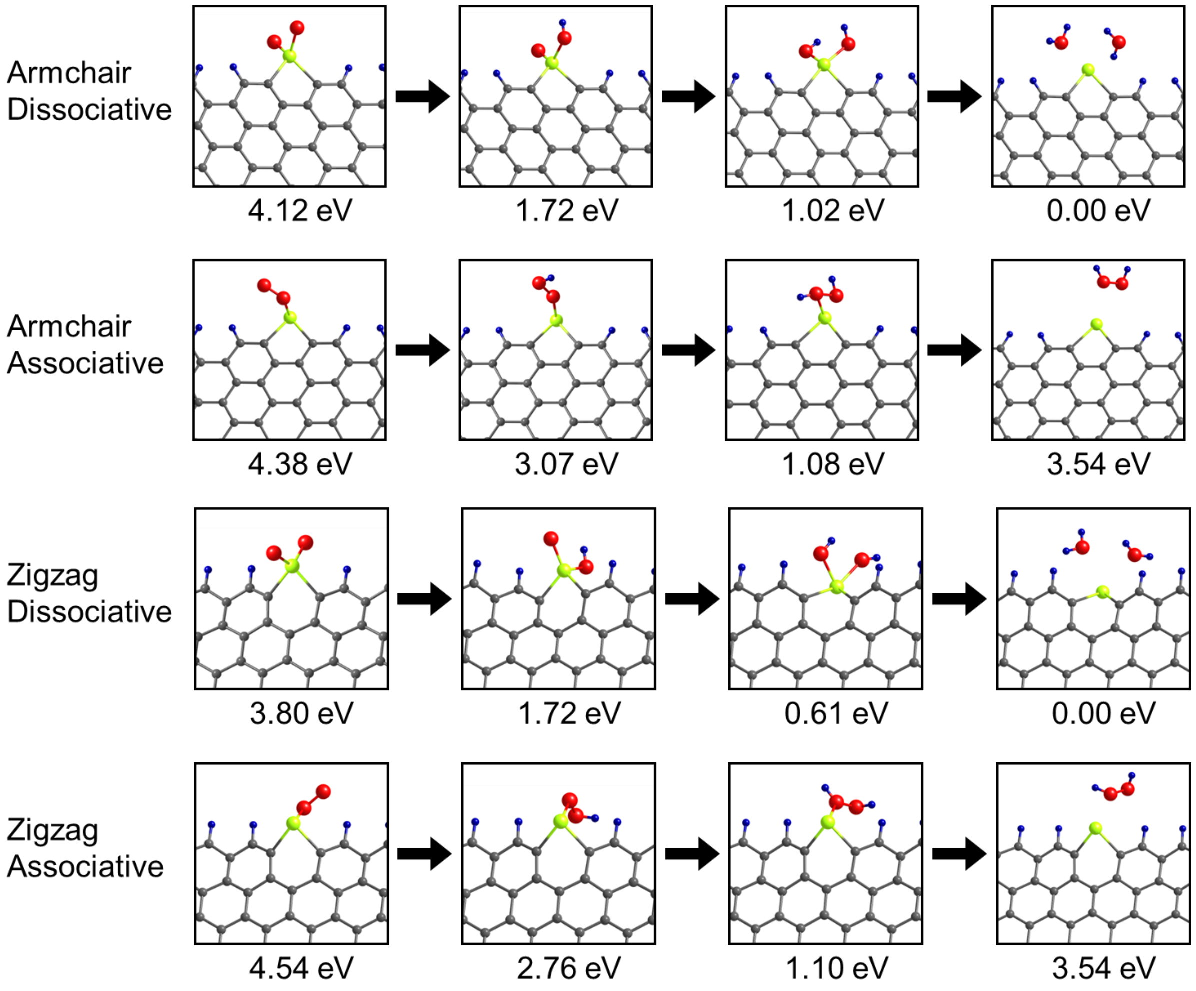

Supplementary Figure 21 | The differences in energy between associative and dissociative process of O_2 at each intermediate step along the reaction path shown in Fig. 5.

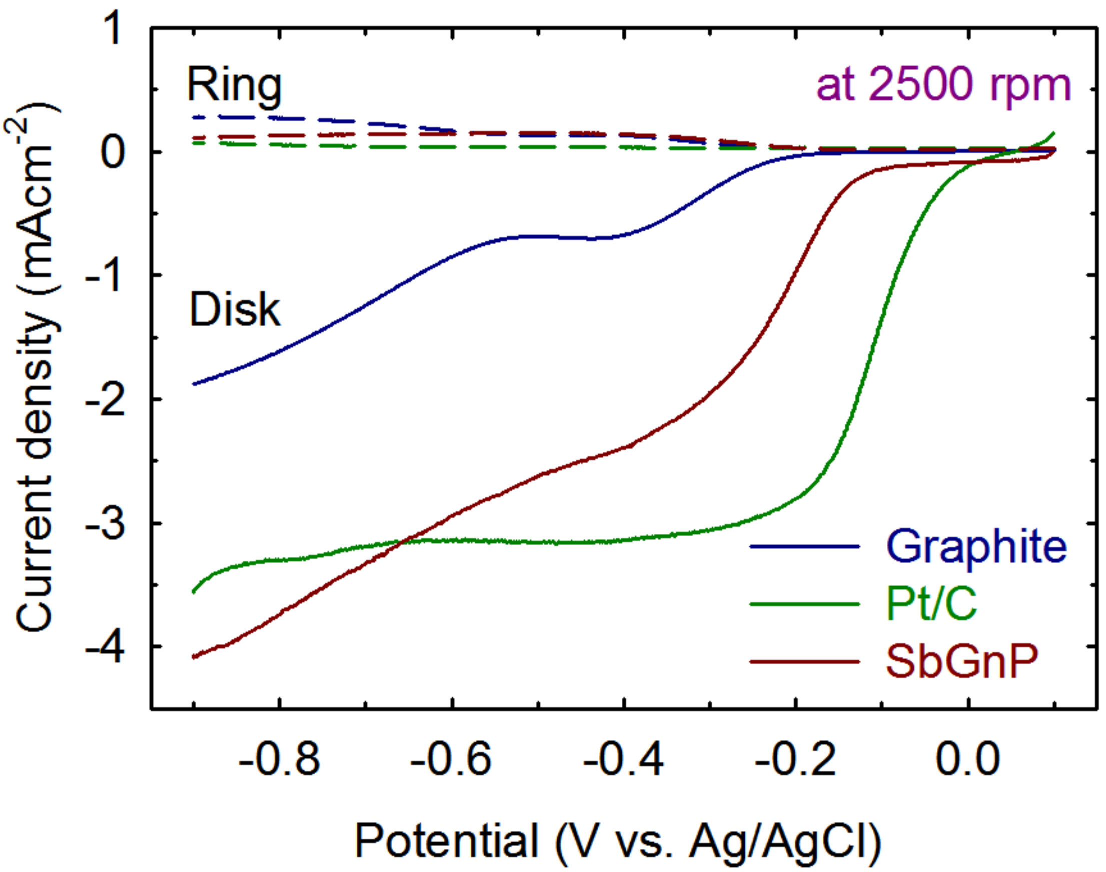

Supplementary Figure 22 | RRDE voltammograms of samples in O_2-saturated 0.1 M aq. KOH solution at a scan rate of 10 mV s^{-1} at rotating rate at 2500 rpm.

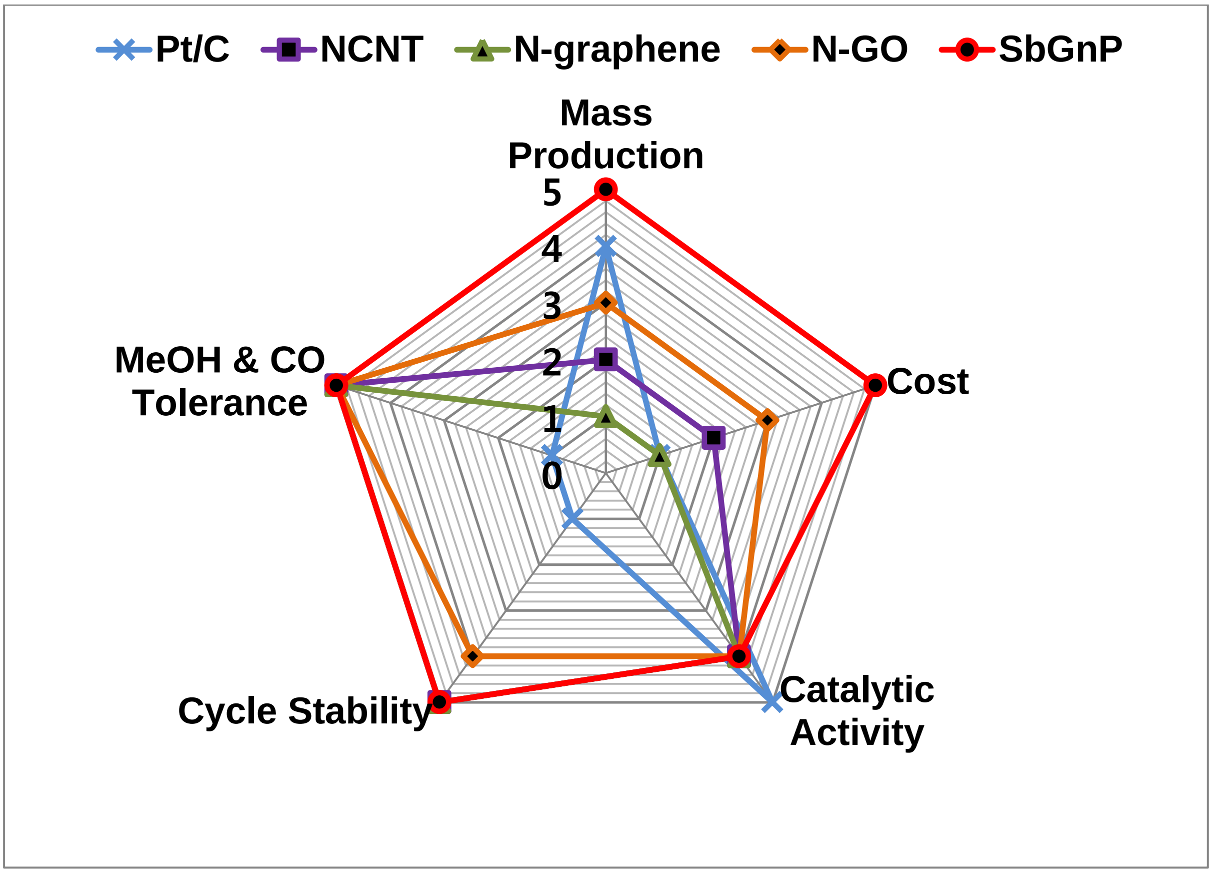

Supplementary Figure 23 | Radar chart on merits of various cathodic electrode materials for oxygen reduction reaction in alkaline media.

Supplementary Table 1 | TGA, EA, EDS and XPS data of the pristine graphite and SbGnPs

Sample	TGA (Char yield at 1000 °C)		Element	EA (wt.%)	EDS (at.%)	XPS (at.%)
	Air	N_2				
Graphite	23.7	99.1	C	99.64	98.80	98.35
			O	0.13	1.20	1.65
SbGnP	10.7	77.2	C	73.44	91.19	82.87
			O	11.95	6.31	15.95
			Cl	-	1.33	-
			H	0.93	-	-
			Sb (wt.%)	(13.68)*	1.17 (10.28)	1.18 (10.53)

* The number in parenthesis is estimated value of weight percent (wt.%).

Supplementary Table 2 | BET surface area, pore volume and pore size of the pristine graphite and SbGnPs

Sample	Surface Area (m^2/g)	Pore Volume (mL/g)	Pore Size (nm)
Graphite	2.78	0.0016	2.27
SbGnP	376.71	0.3857	4.10

Supplementary Table 3 | Capacitance of the pristine graphite, Pt/C and SbGnPs in N_2- and O_2-saturated 0.1 M aq. KOH solution with respect to scan rate

Sample	Specific Capacitance (F/g)					
	Scan Rate	10 mV s^{-1}	20 mV s^{-1}	50 mV s^{-1}	100 mV s^{-1}	200 mV s^{-1}
Graphite	N_2	5.8	4.7	3.7	3.2	2.7
	O_2	32.9	17.5	8.1	4.6	2.6
Pt/C	N_2	47.3	44.0	38.0	33.8	30.8
	O_2	121.3	98.8	79.2	66.0	55.3
SbGnP	N_2	85.1	77.0	68.8	63.9	59.1
	O_2	109.3	99.9	88.1	80.1	72.4

Supplementary Table 4 | The electron transfer number (*n*) for oxygen reduction at different potentials

Sample	Potential (*vs*. Ag/AgCl)		
	-0.4 V	-0.5 V	-0.6 V
Graphite	2.0	2.0	2.0
Pt/C	4.0	4.0	4.0
SbGnP	3.9	4.0	4.0

Supplementary Table 5 | Comparison of cathodic electrode materials for oxygen reduction reaction in alkaline media

Sample / merits	Pt/C	N-doped CNT (CVD)[1]	N-doped graphene (CVD)[2]	N-doped graphene (GO)[3]	Sb-doped GnPs (Ball-mill)
Mass production (Kg)	O	X	X	Δ	O
Production cost	Very high	Very high	Very high	Moderate	Very low
Production process	Simple	Tedious	Tedious	Tedious	Simple
Impurity	Δ	X	X	Δ	X
Dispersibility (Processability)	Moderate	Poor	Poor	Very poor	Good
Structure controllability	O	X	X	X	O
Electron transfer number (n)	n = 4.0 (-0.6 V)	n = 3.9 (-0.3 V)	n = 3.6 - 4.0 (-0.4 ~ -0.8 V)	n = 3.8 (-0.5 V)	n = 4.0 (-0.6 V)
Cycle stability	81.1% after 100,000 cycles	100% after 100,000 cycles	~100% after 200,000 cycles	~100% after 5000 cycles	100% after 100,000 cycles
CO tolerance	X	O	O	-*	O
Methanol tolerance	X	O	O	-*	O

* Not reported.

Supplementary Note 1

Purification procedure. The bulk purity of SbGnPs was investigated using energy-dispersive X-ray (EDX) spectroscopy analysis in TEM. When SbGnPs were simply washed in warm concentrated hydrochloric acid (~37%) at ambient conditions, chromium (Cr) and iron (Fe) were unexpectedly detected in addition to the expected carbon (C), oxygen (O) and antimony (Sb) in the EDS elemental mapping (Supplementary Fig. 1). The elemental contents at different locations could be quantified using the EDX spectra (Supplementary Fig. 2). The dark, transparent spots in the dark-field (DF) TEM image contain only C, O and Sb (Supplementary Fig. 2a). The bright spots consist of Cr and Fe (Supplementary Figs. 2b and 2c), which originated from the stainless steel balls and ball-mill container. The results indicate that the brief acid-washing of SbGnPs in concentrated hydrochloric acid cannot completely remove the metallic residues and free-standing Sb. Further Soxhlet extraction using concentrated hydrochloric acid (~37%) allowed for the complete removal of any metallic residues and free-standing Sb remaining in the SbGnPs.

To further assure reproducibility, another batch of SbGnPs was prepared using the same procedures and work-up conditions. SEM (Supplementary Fig. 5a) with element mapping found no metallic residue, only C (Supplementary Fig. 5b), O (Supplementary Fig. 5c) and Sb (Supplementary Fig. 5d). The corresponding EDX spectra show C, O and Sb together with Cl (Supplementary Fig. 8), which originated from physically adsorbed HCl during the hydrochloric acid work-up. Furthermore, high-resolution TEM (HR-TEM) images of SbGnPs indicate a flake-type morphology (Supplementary Fig. 6a) and a highly ordered structure with a honeycomb lattice in the basal area (Supplementary Fig. 6b). The results indicate that SbGnPs have minimal distortion on their basal plane. A high-angle annular dark field (HAADF) scanning TEM (STEM) image (Supplementary Fig. 6c) with element mappings also indicates the presence of C (Supplementary Fig. 6d), O (Supplementary Fig. 6e) and Sb

(Supplementary Fig. 6f), suggesting that covalent links between C and Sb in SbGnPs are formed. However, there is no trace of Cl, implying that the origin of Cl detected by SEM EDS is due to physically adsorbed HCl[4], which is removed under high vacuum in the TEM analysis. EDS (TEM) analyses at different location indicate that the average content of Sb on SbGnPs was 1.28 at.% (11.47 wt.%, Supplementary Fig. 7).

Supplementary Note 2

DFT Calculations. For computations, we used the Vienna Ab initio Simulation Package (VASP) to calculate the ground state of many electrons system in the frame work of density functional theory[5-8]. The plane-wave basis set with an energy cut-off of 400 eV and the gradient-corrected exchange-correlation potential suggested by Perdew, Burke, and Ernzerhof (PBE-type) were employed[9]. The free energy profile was calculated from the total energy difference, without including the entropy effect of the molecular species. The adsorption structures of the intermediates steps of ORR are calculated through the total energy minimization. The Gibbs free energy of hydroxide ion is calculated with $G(\mathrm{OH}^-) = G(\mathrm{H_2O}) - G(\mathrm{H}^+)$ and $G(\mathrm{H}^+) = \frac{1}{2}G(\mathrm{H_2}) + k_B T \ln[\mathrm{H}^+]$[10].

Supplementary Methods

Materials. Graphite was obtained from Alfa Aesar (Natural, 100 mesh, 99.9995% metals basis, Lot #: 14735) used as received. Antimony (Sb) was purchased from Aldrich Chemical Inc. (Cat.# 266329) and used as received. All other solvents were supplied by Aldrich Chemical Inc. and used without further purification, unless otherwise specified.

Instrumentations. Elemental analysis (EA) was conducted with Thermo Scientific Flash 2000. The field emission scanning electron microscopy (FE-SEM) was performed on FEI Nanonova 230. The high-resolution transmission electron microscopy (HR-TEM) was performed on JEOL JEM-2100F microscope and atomic resolution transmission electron microscopy (AR-TEM) was carried out on Titan Cubed G2 60-300 microscope. The TEM specimen were prepared by dipping carbon micro-grids (Ted Pella Inc., 200 Mesh Copper Grid) into well-dispersed samples in ethanol. Time-of-flight secondary ion mass spectrometry (TOF-SIMS) was carried out with a TOF-SIMS V instrument (ION-TOF GmbH, Germany) using a 10 keV Bi^+ primary ion beam source. The surface area was measured by nitrogen adsorption-desorption isotherms using the Brunauer-Emmett-Teller (BET) method on Micromeritics ASAP 2504N. X-ray photoelectron spectra (XPS) were recorded on a Thermo Fisher K-alpha XPS spectrometer. Themogravimetric analysis (TGA) was conducted on a TA Q200 (TA Instrument) at a heating rate of 10 °C min^{-1} under air and nitrogen atmosphere. Micro-Raman measurements were made with a WiTec Alpha300S system with 532 nm wavelength laser light and a 50× objective. X-Ray diffraction (XRD) patterns were recorded with a Rigaku D/MAZX 2500V/PC with Cu–Kα radiation (35 kV, 20 mA, λ = 1.5418 Å).

Supplementary References

1. Gong, K. P., Du, F., Xia, Z., Durstock, M. & Dai, L. Nitrogen-doped carbon nanotube arrays with high electrocatalytic activity for oxygen reduction. *Science* **323**, 760-764 (2009).
2. Qu, L., Liu, Y., Baek, J.-B. & Dai, L. Nitrogen-doped graphene as efficient metal-free electrocatalyst for oxygen reduction in fuel cells. *ACS Nano* **4**, 1321-1326 (2010)
3. Geng, D. *et al.* High oxygen-reduction activity and durability of nitrogen-doped graphene. *Energy Environ. Sci*. **4**, 760-764 (2011).
4. Hohenberg, P. & Kohn, W. Inhomogeneous electron gas. *Phys. Rev.* **136**, B864 (1964).
5. Filella, M. & May, P. M. Computer simulation of the low-molecular-weight inorganic species distribution of antimony (III) and antimony (V) in natural waters. *Geochim. Cosmochim. Acta* **67**, 4013-4031 (2003).
6. Kresse, G. & Furthmüller, J. Efficient iterative schemes for ab initio total-energy calculations using a plane-wave basis set. *Phys. Rev. B,* **54**, 11169 (1996).
7. Kresse, G. & Furthmüller, J. Efficiency of ab-initio total energy calculations for metals and semiconductors using a plane-wave basis set. *Comput. Mater. Sci.* **6**, 15-50 (1996).
8. Kohn, W. & Sham, L. J. Self-consistent equations including exchange and correlation effects. *Phys. Rev.* **140**, A1133 (1965).
9. Perdew, J. P., Burke, K. & Ernzerhof, M. Generalized gradient approximation made simple. *Phys. Rev. Lett.* 77, 3865 (1996).
10. Dongbin, S., Sinthika, S., Min C., Thapa, R., Noejung, P. *Ab Initio* Study of Thin Oxide-Metal Overlayers as an Inverse Catalytic System of Dioxygen Reduction and Enhanced CO Tolerance. *ACS Catal*. 4, 4074 (2014).
